# Supplementary material for: Policies for type 2 diabetes and non-communicable disease management during the COVID-19 pandemic in Kenya and Tanzania: a desk review and views of decision-makers
Source: BMJ Open. 2024 Mar 25;14(3):e073261. doi: 10.1136/bmjopen-2023-073261 (PMC10966805; doi:10.1136/bmjopen-2023-073261)
Supplement: Supplementary data [file bmjopen-2023-073261supp001.pdf]

Appendices

Appendix A. Search Criteria for Peer-reviewed literature

1. **Diabetes terms:** diabetes, NCDs, disease management, Type 2 diabetes, diabetes mellitus, chronic disease, non-insulin dependent diabetes, ’type 2 diabetes’ OR ‘type II diabetes’ OR ’diabetes mellitus’ OR ‘diabetes’
2. **COVID terms**: covid, coronavirus, COVID-19, SARS-COV-2, severe acute respiratory syndrome coronavirus 2, 2019-nCoV
3. **Healthcare:** healthcare, disruption, impact, health services provision, change, continuity of care, medical care
4. **Policies**: policy, guidelines, measurement/assessment/evaluation, strategies, recommendations, plans, impact, research, outcomes, result, approaches, care continuity, SOPs, procedures, frameworks, policy models, laws, bills, tools
5. **Countries**: Kenya, Tanzania

Appendix B: Table 1: Details of search strategy

**Source**	**Details**
Call for information	A call for information was disseminated via email and social media platforms (WhatsApp) to relevant networks/experts including Ministry of Health officials, National NCD department heads, County health departments, diabetes clinic heads, National COVID-19 task force leads; Technical working groups (national or county), Academic researchers who focus on COVID/diabetes care disruption, and NGOs. We first identified a list of potential stakeholders from relevant networks and sent out the call to them. In the call we requested that they share hard or soft copy documents by email or courier related to the management of T2D and other chronic diseases during COVID-19 or other emergency epidemic or pandemic situations.
Peer reviewed literature databases	We searched peer reviewed literature databases of PubMed/Medline, Embase, Google Scholar, Web of Science, OpenGrey using search criteria to identify articles and grey literature that describe diabetes, COVID-19 and health system responses (*see specific search criteria in Appendix A*). Due to time limitations, this was not a comprehensive systematic review, but rather a rapid review of the literature.
Local/ International resources	Since international health agencies such as the World Health Organization have taken the lead both on COVID-19 responses and on management of NCDs, we reviewed documents from international resources, including the United Nations, World Health Organization, and relevant non-governmental agencies. We included WHO country profiles, WHO documents on COVID, and reports from NGOs working in diabetes in Kenya.
Policy documents/drafts from	We reviewed Kenya's health ministry websites including for all documents associated with national NCD strategies, policy documents and committee reports related to T2D and COVID-19, reports from meetings and events, and standard operating procedures. We searched websites including Ministry of health, and any COVID-specific working group, etc. for policies,

relevant national ministries	guidelines, measurement/assessment/evaluation, strategies, recommendations, plans, impact, research, outcomes, result, approaches, care continuity, SOPs, procedures, frameworks, policy models, laws, bills, tools.
Internet search	Given the extensive use of the internet to communicate during the pandemic, we conducted an internet search using google, Google Scholar and other search engines to identify patient support groups, diabetes association's/message boards, newspaper/media articles regarding T2D and COVID.
Social Media	Finally, given the speed with which governments needed to react to COVID-19 developments, many governmental individuals and offices disseminated information to the public on social media. We reviewed twitter pages of the country's Ministry of Health and other high-profile individuals/offices, presidential speeches or other speeches on social media and national NCD groups for information about T2D and COVID-19.

Appendix C: Key Informant Interview guide for policy actors working in non-communicable diseases (NCDs).

GECO Study

Project Title: Healthcare and Socio-economic Impacts of COVID-19 on Patients with type 2 Diabetes in Kenya

Key Informant Interview (KII) guide – Policy Actors working in non-communicable diseases (NCDs).

AFRICAN POPULATION AND HEALTH RESEARCH CENTRE

These are the interviews with policy actors and decision makers working in non-communicable diseases and other related fields. The objective is to identify policy gaps in Kenya to inform policy change, priority setting and action for T2D management during COVID-19

Introduction and Warm up

Moderator Note: Moderator to read out from written informed consent form and obtain signature/ thumbprint)

- The moderator introduces him/herself and explains the purpose of the study.
- Collects relevant information on the Participant Description Form (PDF) during screening.
- The moderator explains that the discussion is open, not an exam and there is no right or wrong answer. The facilitator/moderator should explain to the respondent that the information given by the participant is confidential.
- Encourage the respondent to give honest opinions.
- Explain the use of the recorder.
- Start tape recording if consent is granted: (Moderator to switch recorder on)

Remember to introduce each section to the participant to prepare them for the questions to be asked.

This study aims to identify policy gaps in Kenya to inform policy change, priority setting and action for T2D management during COVID-19. In particular, we would like to know what policies/guidelines have been developed during COVID-19, who and which sectors have been involved in the policy development and implementation, challenges in the formulation and implementation, gaps that still exist and recommendations. We are interested in higher level policies such as laws, regulations, national strategic plans, directives as well as lower level guidelines, action plans, SOPs etc. related to T2DM during COVID-19.

Section A: General response of governments to COVID-19

1. Briefly tell me what the government's response has been towards the management of NCDs including type 2 diabetes during COVID-19 in Kenya?

Section B: Policy formulation during COVID-19

2. Please tell me about the policies and guidelines that you are aware of that were **formulated** during COVID-19 to manage people with type 2 diabetes

   Probes:

   Context

   a) What was the rationale for the formulation of the policy/guidelines mentioned? (context)
      i. What is it about the COVID-19 situation that necessitated the development of the policy/ guideline?
      ii. Was there any research that informed the need to update/revise the pre-existing NCD/diabetes policies/guidelines? Please explain.

   Content

   b) What do the policies/guidelines include? (content)
      i. Were the provisions (content) in the policy adequate to manage type 2 diabetes during COVID-19? If not, what else do you think should be added?
      ii. Was the content in the policy/guideline informed by research findings?

   Actors and Process

   c) Were you involved in the formulation of these policies you have mentioned? (actors)
   d) What was your role in the formulation?
   e) Who else or what other sectors were involved in the formulation of the policy or guidelines?
      i. Who led the process of formulating the type 2 diabetes policies or guidelines?
      ii. What was the role of the sectors involved?
      iii. Who else should have been involved in the formulation?
         i. Were healthcare providers involved in the formulation?
   f) What were the advantages of having these policies/guidelines in place during COVID-19?
   g) What were the challenges encountered during the formulation process of the policy/ guidelines for type 2 diabetes?

3. What resources were available for the **formulation** of these policies/guidelines?

   Probes:

   a) Type of resources available? (funding, human resource, experts in the field)

Section C: Policy implementation during COVID-19

4. To what extent have the policies/guidelines developed during COVID-19 been **implemented**

Probes:

a) Were you involved in their implementation? (actors)
b) What was your role in the implementation?
c) Who else or what other sectors were involved in the implementation of the policy/guidelines?
   i. Who led the implementation of the policies/guidelines?
   ii. What was the role of the sectors involved?
   iii. Who else should have been involved in the implementation? Why?
d) What were the advantages of implementing these policies/guidelines during COVID-19?
e) What were the challenges encountered during the implementation of the policy/ guidelines?

5. What resources were available for the **implementation** of these policies/guidelines?

Probes:

a) Type of resources available? (funding, human resource, experts in the field)
b) Were the resources adequate? What was missing? Elaborate

6. What mechanisms were used to disseminate the new policies/guidelines on the management of type 2 diabetes during COVID-19?

Probes:

a) Types of mechanisms? (trainings, directives shared etc?)
b) How does the government ensure that the policies/guidelines are actually being used?

Section D: Recommendations

7. What gaps still exist in the management of type 2 diabetes during COVID-19?
   a) How can they be addressed to avoid future disruption in care during an emergency/pandemic situation?
8. In your opinion, how best can the Kenyan health system **strengthen its response** towards chronic disease management in the event of an emergency/pandemic situation?
9. What would you recommend to ensure the continuity of chronic care management (including type 2 diabetes) during an emergency/pandemic situation?
10. Is there anything else you would like to add with regard to chronic care management including diabetes in Kenya that we have not discussed?

We have come to the end of the interview***. [Thank the participant for participating in the interview]***

*****END*****

Appendix D: Description of the documents included in the desk review for Kenya and Tanzania

Type of Document	Name of Document	Country	Dates	Content Description
1. Policy [12]	Tanzania Health Policy 2007	Tanzania	2007	The policy provides direction on the development of NCD guidelines that will provide guidance on the provision of health service, equity in service provision, capacity building, and raising awareness on the management and prevention of NCDs.
2. Policy [13]	Kenya Health Policy 2014-2030	Kenya	2014	The document provides overall directions to ensure significant improvement in the overall status of health in Kenya, the country's long-term development agenda, Vision 2030 and global commitments including halting and reversing the rising burden of NCDs in the country.
3. National Framework [14]	National Strategy for the Prevention and Control of NCDs 2015 -2020	Kenya	2015 - 2020	The document emphasizes an integrated approach towards addressing NCDs in order to reduce the preventable burden, avoidable morbidity, mortality, risk factors, and costs due to NCDs. It provides evidence based NCD prevention and control interventions to ensure optimal health throughout the life course for sustainable socioeconomic development.
4. National Framework [15]	Health Sector Strategic Plan (HSSP IV)	Tanzania	2015-2020	The document clarifies the importance of integrating NCD clinics into the health care system to enhance access to care. It also describes NCD prevention and early detection of the NCD and start of medication.
5. National framework [16]	Strategic and action plan for the prevention and control of NCDs in Tanzania 2016 – 2020	Tanzania	2016-2020	The document advocates for NCD prevention and control at a national level and it emphasizes strengthening leadership, governance, multisectoral collaboration, and accountability for the prevention and control of NCDs and promoting preventive curative and rehabilitative services.

6. Guideline [17]	National guideline of clinical management and infection prevention and control of novel coronavirus (COVID-19)	Tanzania	April 2020	This guideline educates the public on COVID-19 preventive measures and emphasizes the provision of accurate information for people with NCDs to protect them from COVID-19.
7. Guideline [18]<br><br>8. Guideline [19]	Interim Guidelines on Management of COVID-19 in Kenya<br><br>**Updated version:** Guidelines on Case Management of COVID-19 in Kenya	Kenya	April 2020<br><br>July, 2021	The guidelines combine both preventive and clinical management of diseases during COVID-19. The updated version (2021) contains the latest guidelines for the clinical management of COVID-19 and includes case definitions, infection prevention and control, diagnosis and management of COVID-19, and ending isolation for COVID-19 patients.
9. Letter [20]	Non-Communicable Diseases Clinic During COVID-19 Outbreak	Kenya	Mid-April 2020	This document is a national directive for NCD clinics to remain operational in all counties during the COVID-19 pandemic.
10. Guideline [21]	Guidance on the provision of NCD and mental health services in the context of the COVID-19 outbreak in Tanzania	Tanzania	May 2020	This guideline targets strengthening NCD service provision during COVID-19 including capacity building for health workers, health promotion for NCD/T2D patients, health facility re-structuring for COVID-19 prevention, and access to care and continuity of essential health services.
11. Guideline [22]<br><br>12. Guidelines [23]	Interim Guidance on Continuity of Essential Health Services during the Covid-19 Pandemi**c.**<br><br>**Updated version:**<br>Guidance on Continuity of Essential Health Services	Kenya	May 2020<br><br>July 2020	These documents provide guidance for healthcare providers on immediate actions that should be considered to reorganise the health system to ensure continuity of health services during the COVID-19 pandemic for all services including NCD services.

	during the COVID-19 Pandemic			
13. Guideline [24]	Interim Guidance on Provision of Services for NCDs during the COVID-19 Pandemic	Kenya	July 2020	The document highlights specific COVID-19 related challenges on NCDs including severity among high-risk persons, COVID-19 actions/responses jeopardising access to care and disrupting lifestyle approaches, disruption of funding and supplies, and the general management of NCDs.
14. Report [25]	Flash appeal for COVID-19 Tanzania	Tanzania	July – December 2020	The document discusses strengthening capacity building for health workers, ensuring continued mentorship on NCD and COVID-19 health management, and disseminating information materials and guidelines. It also recommends health promotion and public health intervention strategies for quality-of-life improvement for those with complications associated with NCDs.
15. Policy Brief [26]	Tanzania NCD Alliance (TANCDA) policy brief on the inclusion of NCDs on universal health coverage in Tanzania	Tanzania	March 2021	The document recommends various actions to ensure access to quality NCD care, appropriate health financing strategies, multisectoral collaboration on prevention and control of NCDs, and health awareness creation during the COVID-19 pandemic.
16. Policy [27]	Kenya Emergency Medical Care Policy 2020 – 2030	Kenya	July 2021	This document was developed to provide guidance including necessary structures, resources, regulations and standards needed to establish an emergency medical care system in Kenya. It aims to reduce morbidity and mortality caused by medical emergencies, including those emergency conditions caused by NCDs.
17. National framework [28]	National Strategic Plan for the Prevention and Control of Non-Communicable Disease 2021/22 - 2025/26	Kenya	August 2021	The document provides direction on strengthening sectoral and multi-sectoral coordination, leadership and governance for prevention and response to NCDs at all levels. It highlights the importance of sustainable NCD management, promotion of NCD research, and strengthening surveillance, monitoring and evaluation of NCDs to inform decision making and health planning.
