# Supplementary material for: Policies for type 2 diabetes and non-communicable disease management during the COVID-19 pandemic in Kenya and Tanzania: a desk review and views of decision-makers
Source: BMJ Open. 2024 Mar 25;14(3):e073261. doi: 10.1136/bmjopen-2023-073261 (PMC10966805; doi:10.1136/bmjopen-2023-073261)
Supplement: Author's manuscript [file bmjopen-2023-073261.draft_revisions.pdf]

BMJ Open is committed to open peer review. As part of this commitment we make the peer review history of every article we publish publicly available.

When an article is published we post the peer reviewers' comments and the authors' responses online. We also post the versions of the paper that were used during peer review. These are the versions that the peer review comments apply to.

The versions of the paper that follow are the versions that were submitted during the peer review process. They are not the versions of record or the final published versions. They should not be cited or distributed as the published version of this manuscript.

BMJ Open is an open access journal and the full, final, typeset and author-corrected version of record of the manuscript is available on our site with no access controls, subscription charges or pay-per-view fees (http://bmjopen.bmj.com).

If you have any questions on BMJ Open's open peer review process please email info.bmjopen@bmj.com

BMJ Open

Policies for chronic disease management during the COVID-19 pandemic in Kenya and Tanzania: a desk review and views of decision makers.

Journal:	*BMJ Open*
Manuscript ID	bmjopen-2023-073261
Article Type:	Original research
Date Submitted by the Author:	28-Feb-2023

Complete List of Authors:	Mohamed, Shukri; African Population and Health Research Center<br>Kisia, Lyagamula; African Population and Health Research Center<br>Mashiashi, Irene; Ifakara Health Institute<br>Mair, Frances; University of Glasgow, General Practice and Primary Care<br>Wisdom, Jennifer P.; Wisdom Consulting<br>Bunn, Christopher; University of Glasgow College of Social Sciences, Institute of Public Health<br>Gray, Cindy; University of Glasgow, School of Social and Political Sciences; University of Glasgow<br>Kibe , Peter M. ; African Population and Health Research Center<br>Sanya, Richard; African Population and Health Research Center, Health and Wellbeing<br>Karugu, Caroline; African Population and Health Research Center<br>Mtenga, Sally M.; Ifakara Health Institute<br>Binyaruka, Peter; Ifakara Health Institute<br>Asiki, G; African Population and Health Research Center

Keywords:	COVID-19, Health policy < HEALTH SERVICES ADMINISTRATION & MANAGEMENT, PUBLIC HEALTH

SCHOLARONE™
Manuscripts

I, the Submitting Author has the right to grant and does grant on behalf of all authors of the Work (as defined in the below author licence), an exclusive licence and/or a non-exclusive licence for contributions from authors who are: i) UK Crown employees; ii) where BMJ has agreed a CC-BY licence shall apply, and/or iii) in accordance with the terms applicable for US Federal Government officers or employees acting as part of their official duties; on a worldwide, perpetual, irrevocable, royalty-free basis to BMJ Publishing Group Ltd ("BMJ") its licensees and where the relevant Journal is co-owned by BMJ to the co-owners of the Journal, to publish the Work in this journal and any other BMJ products and to exploit all rights, as set out in our licence.

The Submitting Author accepts and understands that any supply made under these terms is made by BMJ to the Submitting Author unless you are acting as an employee on behalf of your employer or a postgraduate student of an affiliated institution which is paying any applicable article publishing charge ("APC") for Open Access articles. Where the Submitting Author wishes to make the Work available on an Open Access basis (and intends to pay the relevant APC), the terms of reuse of such Open Access shall be governed by a Creative Commons licence – details of these licences and which Creative Commons licence will apply to this Work are set out in our licence referred to above.

Other than as permitted in any relevant BMJ Author's Self Archiving Policies, I confirm this Work has not been accepted for publication elsewhere, is not being considered for publication elsewhere and does not duplicate material already published. I confirm all authors consent to publication of this Work and authorise the granting of this licence.

For peer review only

Policies for chronic disease management during the COVID-19 pandemic in Kenya and Tanzania: a desk review and views of decision makers.

Authors:
Shukri F. Mohamed[1*]
Lyagamula Kisia[1]
Irene Mashiashi[2]
Frances S Mair[3]
Jennifer P. Wisdom[4]
Christopher Bunn[5]
Cindy Gray[5]
Peter Kibe[1]
Richard Sanya[1]
Caroline Karugu[1]
Sally Mtenga[2]
Peter Binyaruka[2]
Gershim Asiki[1]

Author Affiliations
[1] Chronic Disease Management Unit, African Population and Health Research Center (APHRC), Nairobi, Kenya
[2] Ifakara Health Institute, Dar-es-Salaam, Tanzania.
[3] School of Health and Wellbeing, University of Glasgow, UK
[4] Wisdom Consulting, New York, NY, USA
[5] School of Social and Political Sciences, University of Glasgow, UK.

*Corresponding author: Shukri F. Mohamed – smohamed@aphrc.org

Word count - 6161

For peer review only

Abstract

Background: The COVID-19 pandemic caused disruptions in care that adversely affected the management of non-communicable diseases (NCDs) globally. Countries have responded in various ways to support people with NCDs during the pandemic. This study aimed to identify policy gaps, if any, in the management of NCDs, particularly diabetes, during COVID-19 in Kenya and Tanzania to inform recommendations for priority actions for NCD management during any future similar crises.

Methods: We undertook a desk review of pre-existing and newly developed national frameworks, policy models and guidelines for addressing NCDs including type 2 diabetes. This was followed by 13 key informant interviews with stakeholders involved in NCD decision making: six in Kenya; seven in Tanzania. Thematic analysis used to analyse the documents.

Results: Seventeen guidance documents identified (Kenya = 10; Tanzania = 7). These included pre-existing and/or updated policies/strategic plans, guidelines, a letter, a policy brief and a report. Neither country had comprehensive policies/guidelines to ensure continuity of NCD care before the COVID-19 pandemic. However, efforts were made to update pre-existing documents and several more were developed during the pandemic to guide NCD care. Some measures were put in place during the COVID-19 period to ensure continuity of care for patients with NCDs such as longer supply of medicines. Inadequate attention was given to monitoring and evaluation and implementation issues.

Conclusion: Kenya and Tanzania developed and updated some policies/guidelines to include continuity of care in emergencies. However, there were gaps in the documents and between policy/guideline documents and practice. Health systems need to establish disaster preparedness plans that integrate attention to NCD care to enable them to better handle severe disruptions caused by emergencies such as pandemics. Such guidance needs to include contingency planning to enable adequate resources for NCD care and must also address evaluation of implementation effectiveness.

Word count: 297

Key words: Non-communicable diseases, NCDs, diabetes, East Africa, care continuity, care disruption, Sub-Saharan Africa

For peer review only

What is already known?

- The COVID-19 pandemic caused disruptions in care that adversely affected the management of non-communicable diseases (NCDs) globally.
- Countries introduced various strategies to support people with NCDs including diabetes during this pandemic.

What are the new findings?

- This review revealed that prior to the COVID-19 pandemic, both countries did not have comprehensive policies/guidelines that addressed the management of NCDs during pandemic/emergency situations.
- Several policies and guidelines were developed during the COVID-19 pandemic and measures were put in place to address the needs of patients with NCD and T2DM but inadequate attention was given to assessing implementation effectiveness.

What do the new findings imply?

- Public health emergencies will continue to occur; therefore, there is a strong need to develop disaster preparedness plans that include attention to NCD care, to better cope with future emergency situations and to strengthen health systems' resilience to multiple shocks.
- Health systems need to be better prepared to handle disruptions in care by establishing emergency preparedness plans and, adequately resourcing implementation efforts.

For peer review only

For peer review only - http://bmjopen.bmj.com/site/about/guidelines.xhtml

Introduction

The COVID-19 pandemic caused disruptions in care that adversely affected the management of non-communicable diseases (NCDs) globally. Government restrictions implemented in various countries to curb the spread of COVID-19 such as lockdown directives and mobility restrictions impacted management of NCDs by individuals and health systems.[1] A rapid assessment conducted by the World Health Organisation (WHO) across 163 countries revealed that NCD health services including rehabilitation services, hypertension management, and diabetes management, were partially or completely disrupted by the pandemic.[2] These disruptions are likely to have ongoing negative impacts on the management of chronic diseases and progress toward achieving Sustainable Development Goal (SDG) targets. Studies reported that patients with NCDs such as hypertension and diabetes were more likely than those without these comorbidities to be affected by COVID-19 and suffer worse health outcomes if infected.[3 4]

Despite most countries prioritizing services for four major NCDs (diabetes, hypertension, cancer, and cardiovascular disease) during the COVID-19 pandemic,[2] maintaining routine care for NCDs is a major challenge due to barriers at various levels of healthcare systems. Patients with Type 2 diabetes (T2D) may need continuous and regular blood glucose monitoring and optimal compliance with ongoing drug treatment.[5] Healthcare providers reported that diabetes was the most affected condition by the reduction in healthcare resources.[1] The WHO assessment found that 66% of countries surveyed included the continuity of NCD services in the national COVID-19 plans.[2]

Countries have used various strategies to support people with NCDs including diabetes during this pandemic,[6] primarily switching from routine care visits to virtual healthcare visits and communications.[1] Despite these adjustments in service delivery for NCDs, most global healthcare resources focused on the COVID-19 emergency response,[7] particularly in Sub-Saharan Africa (SSA) where healthcare personnel and health resources are inadequate.[8] This resource reallocation disrupted care for patients with NCDs.[9]

In this review, we synthesise policy evidence regarding T2D management during COVID-19 and identified policy gaps, if any, in management of T2D and other NCDs to inform T2D management actions during COVID-19 in Kenya and Tanzania. Our research questions:

(1) Are there pre-existing national frameworks, policy models (priorities, strategies, and directions) and guidelines (instructions) for addressing T2D /NCDs? If so, did they cover

For peer review only - http://bmjopen.bmj.com/site/about/guidelines.xhtml

pandemic/emergency situations? How have existing frameworks/models (includes formal framework/models and informal statements) been modified for pandemic/emergency situations (e.g., COVID-19) OR how have framework and policy models been created during COVID-19 (including those in development)?

(2) What do the national frameworks, policy models or guidelines tend to include with regards to priority setting (access to care and medical supplies, resource allocation and monitoring and evaluation tools) for management of T2D /NCDs in pandemic/emergency situations?

(3) What are the policy/guideline gaps for the management of T2D during the COVID-19 pandemic (e.g., addressing existing disparities such as socioeconomic status and urban/rural status, addressing health service staff, financial resources for health management, implementation, communication/dissemination), and what are the opportunities to improve the management of T2D/NCDs?

Methods

We conducted a systematic search of policy documents and guiding frameworks on NCDs with a particular focus on T2D in Kenya and Tanzania from October 2021 to March 2022. Our review included pre-existing national frameworks, policy models or guidelines, noting whether these had been modified to include pandemic/emergency situations, as well as new ones developed during the COVID-19 pandemic. Identified documents were assessed to ascertain policy/guideline gaps, if any, and opportunities to improve the management of T2D or other NCDs during periods where there are disruptions in care such as the COVID-19 pandemic. While the primary interest was T2D, we also included policy documents which broadly covered NCDs and reviewed their applicability to T2D.

This review was further supplemented by Key Informant Interviews (KIIs)[10] conducted between March and August 2022. The aim of the interviews was to understand how the Kenyan and Tanzanian governments addressed the management of NCDs including T2D during COVID-19, with specific focus on: policy formulation during COVID-19, policy implementation during COVID-19, and gaps noted in the management of NCDs and T2D during COVID-19. Data from the review and the key informant's interview were analyzed separately and then triangulated.

1. **Document review**

For peer review only - http://bmjopen.bmj.com/site/about/guidelines.xhtml

Eligibility criteria

For inclusion in this review documents had to meet four criteria: (1) policy frameworks, policy statements, government statements/pronouncements, publications, emergency preparedness plans, strategies, laws, and operational guidelines for diabetes during COVID-19 (this also included policies under development); (2) specifically mentions NCDs or diabetes or pandemic/emergency or COVID-19; (3) printed in English/Kiswahili; and (4) references Kenya or Tanzania.

Search Strategy

A team of seven researchers (Kenya = 4; Tanzania = 3) with backgrounds in medicine, public health, and social sciences conducted the searches and reviewed the documents. All received training on conducting desk reviews. The team coordinator assigned each researcher a different database to search for relevant documentation.

We searched using key text words such as 'Diabetes' AND 'COVID-19', AND 'Healthcare' AND 'Policies' AND 'Kenya' OR 'Tanzania'. Appendix 1 describes the specific terms used in the search. Using these terms, researchers searched scientific databases (PubMed/Medline, Embase, and Web of Science), Ministry of Health (MOH) websites, local/ international resources such as the WHO, and government social media such as the MOH Twitter and Instagram accounts, supplemented with Google searches. We also contacted existing networks and experts in the NCD field through a call for information which was sent via email and WhatsApp groups to ask for any other relevant policy documents.

All retrieved documents were catalogued in an online excel database which facilitated a standardised approach to data extraction from each document. The document characterisation included a unique document number for tracking the title of the document, source of the document (e.g., social media, MOH), and basic information about the document (e.g., year document was produced). The team held weekly "Coding clinics" to ensure consistency in coding of the included documents.

2. <u>Key informant interviews (KII)</u>

Design and participants

For peer review only

For peer review only - http://bmjopen.bmj.com/site/about/guidelines.xhtml

The results from the document review were used to inform development of the interview guides for the KIIs. In both Kenya and Tanzania, key informants (KIs) were purposively selected based on their experiences and participation in NCD related policy formulation and implementation processes.

Data collection procedures

A semi-structured interview guide was used to explore and understand: a) what policies/guidelines/frameworks had been developed during COVID-19; b) who and which sectors had been involved in the policy development and implementation; c), challenges in the formulation and implementation; d) gaps that still exist; and e) recommendations.

Interviews were conducted by two experienced qualitative researchers who were familiar with the NCDs/T2D policy documents. Interviews were conducted in English in Kenya and in Kiswahili in Tanzania. Participants chose between in-person or remote interviews, to ensure convenience and mitigate risk. The interviews lasted approximately 45 minutes. All interviews were audio-recorded and complemented by handwritten notes.

Data management and analysis

We used content analysis[11] to identify the nature of included documents, the content of the documents, what the documents included regarding priority setting (access to care and medical supplies, resource allocation and monitoring and evaluation tools) and the gaps and opportunities within each document. The information from the desk review was triangulated with the key informants' information to ensure a more in-depth understanding of the synthesised documents and to provide further insights into policy recommendations for NCD management for future emergency situations. The transcripts were coded manually in Word. Two independent researchers, experienced in qualitative data analysis, double coded two transcripts in each country to ensure coding consistency. We obtained a percent agreement of 82%. Disagreements were resolved by rereading the transcripts, discussion and a consensus was reached. In Tanzania, on request from the Tanzania NCD Alliance, a further narrative synthesis of the findings was shared with an NCD expert to identify if there was any important information that was left out, misinterpreted, or missed during the search.

Patient and public involvement

For peer review only - http://bmjopen.bmj.com/site/about/guidelines.xhtml

Patients or the public were not involved in the design, or conduct, or reporting, or dissemination of the research. Results of the study were presented to study participants and stakeholders.

Results

Figure 1 describes the search results. After excluding duplicates and records that were not relevant based on title and abstract screening, we identified 34 policy/guidance documents for full text review. After full text review, 17 documents were further excluded because they did not contain information on managing T2D in a pandemic or emergency situation, leaving 17 documents that were included in this review.

Among the included documents, ten were from Kenya and seven were from Tanzania. In Kenya, we found three interim guidelines, two updated guidelines and a letter from the Director General of Health. In Tanzania, two modified guidelines, one policy brief, and one report were included in the review. Each country had one pre-existing national health policy, one pre-existing NCD strategic plan, and an updated NCD strategic plan.

In total, 13 key informant interviews (Tanzania = 7; Kenya = 6) were conducted with policy actors between March and August 2022. All participants were in the health sector and had different backgrounds in medicine, epidemiology, strategic management, and nutrition. Most of the participants served in various capacities within the MOH. Table 1 provides the characteristics of the KIs interviewed.

Table 1: Characteristics of Key Informants

	Kenya	Tanzania	Total
**Category**			
Government	6	4	**10**
Bilateral organizations/NGOs	0	3	**3**
**Years served in that capacity**			
Less than 1 year	3	0	**3**
1 – 5 years	3	7	**10**
**Total**	**6**	**7**	**13**

The findings are presented by research question.

For peer review only - http://bmjopen.bmj.com/site/about/guidelines.xhtml

Table 2: Description of the documents included in the desk review for Kenya and Tanzania

Type of Document	Name of Document	Country	Dates	Content Description
1. Policy [12]	Tanzania Health Policy 2007	Tanzania	2007	The policy provides direction on the development of NCD guidelines that will provide guidance on the provision of health service, equity in service provision, capacity building, and raising awareness on the management and prevention of NCDs.
2. Policy [13]	Kenya Health Policy 2014-2030	Kenya	2014	The document provides overall directions to ensure significant improvement in the overall status of health in Kenya, the country's long-term development agenda, Vision 2030 and global commitments including halting and reversing the rising burden of NCDs in the country.
3. National Framework [14]	National Strategy for the Prevention and Control of NCDs 2015 -2020	Kenya	2015 - 2020	The document emphasizes an integrated approach towards addressing NCDs in order to reduce the preventable burden, avoidable morbidity, mortality, risk factors, and costs due to NCDs. It provides evidence based NCD prevention and control interventions to ensure optimal health throughout the life course for sustainable socioeconomic development.
4. National Framework [15]	Health Sector Strategic Plan (HSSP IV)	Tanzania	2015-2020	The document clarifies the importance of integrating NCD clinics into the health care system to enhance access to care. It also describes NCD prevention and early detection of the NCD and start of medication.
5. National framework [16]	Strategic and action plan for the prevention and control of NCDs in Tanzania 2016 – 2020	Tanzania	2016-2020	The document advocates for NCD prevention and control at a national level and it emphasizes strengthening leadership, governance, multisectoral collaboration, and accountability for the prevention and control of NCDs and promoting preventive curative and rehabilitative services.

For peer review only - http://bmjopen.bmj.com/site/about/guidelines.xhtml

6. Guideline [17]	National guideline of clinical management and infection prevention and control of novel coronavirus (COVID-19)	Tanzania	April 2020	This guideline educates the public on COVID-19 preventive measures and emphasizes the provision of accurate information for people with NCDs to protect them from COVID-19.
7. Guideline [18]<br><br>8. Guideline [19]	Interim Guidelines on Management of COVID-19 in Kenya<br><br>**Updated version:** Guidelines on Case Management of COVID-19 in Kenya	Kenya	April 2020<br><br>July, 2021	The guidelines combine both preventive and clinical management of diseases during COVID-19. The updated version (2021) contains the latest guidelines for the clinical management of COVID-19 and includes case definitions, infection prevention and control, diagnosis and management of COVID-19, and ending isolation for COVID-19 patients.
9. Letter [20]	Non-Communicable Diseases Clinic During COVID-19 Outbreak	Kenya	Mid-April 2020	This document is a national directive for NCD clinics to remain operational in all counties during the COVID-19 pandemic.
10. Guideline [21]	Guidance on the provision of NCD and mental health services in the context of the COVID-19 outbreak in Tanzania	Tanzania	May 2020	This guideline targets strengthening NCD service provision during COVID-19 including capacity building for health workers, health promotion for NCD/T2D patients, health facility re-structuring for COVID-19 prevention, and access to care and continuity of essential health services.
11. Guideline [22]<br><br>12. Guidelines [23]	Interim Guidance on Continuity of Essential Health Services during the Covid-19 Pandemi**c.**<br><br>**Updated version:** Guidance on Continuity of Essential Health Services during the COVID-19	Kenya	May 2020<br><br>July 2020	These documents provide guidance for healthcare providers on immediate actions that should be considered to reorganise the health system to ensure continuity of health services during the COVID-19 pandemic for all services including NCD services.

For peer review only - http://bmjopen.bmj.com/site/about/guidelines.xhtml
For peer review only

	Pandemic			
13. Guideline [24]	Interim Guidance on Provision of Services for NCDs during the COVID-19 Pandemic	Kenya	July 2020	The document highlights specific COVID-19 related challenges on NCDs including severity among high-risk persons, COVID-19 actions/responses jeopardising access to care and disrupting lifestyle approaches, disruption of funding and supplies, and the general management of NCDs.
14. Report [25]	Flash appeal for COVID-19 Tanzania	Tanzania	July – December 2020	The document discusses strengthening capacity building for health workers, ensuring continued mentorship on NCD and COVID-19 health management, and disseminating information materials and guidelines. It also recommends health promotion and public health intervention strategies for quality-of-life improvement for those with complications associated with NCDs.
15. Policy Brief [26]	Tanzania NCD Alliance (TANCDA) policy brief on the inclusion of NCDs on universal health coverage in Tanzania	Tanzania	March 2021	The document recommends various actions to ensure access to quality NCD care, appropriate health financing strategies, multisectoral collaboration on prevention and control of NCDs, and health awareness creation during the COVID-19 pandemic.
16. Policy [27]	Kenya Emergency Medical Care Policy 2020 – 2030	Kenya	July 2021	This document was developed to provide guidance including necessary structures, resources, regulations and standards needed to establish an emergency medical care system in Kenya. It aims to reduce morbidity and mortality caused by medical emergencies, including those emergency conditions caused by NCDs.
17. National framework [28]	National Strategic Plan for the Prevention and Control of Non-Communicable Disease 2021/22 - 2025/26	Kenya	August 2021	The document provides direction on strengthening sectoral and multi-sectoral coordination, leadership and governance for prevention and response to NCDs at all levels. It highlights the importance of sustainable NCD management, promotion of NCD research, and strengthening surveillance, monitoring and evaluation of NCDs to inform decision making and health planning.

For peer review only - http://bmjopen.bmj.com/site/about/guidelines.xhtml

Research Question 1: What national frameworks, policy models and guidelines for addressing Type 2 Diabetes/NCDs pre-existed and which ones were modified or developed during the COVID-19 pandemic?

Pre-existing documents

There were five documents in Kenya and Tanzania that addressed NCDs or emergency situations prior to the COVID-19 pandemic but none specifically provided guidance on NCD preparedness in an emergency situation. Table 2 provides a brief description of the included documents. The pre-existing documents include: the Tanzania health policy 2007,[12] the Kenya health policy 2014,[13] the Kenya NCD strategic plan 2015-2020,[14] the Tanzania Health Sector Strategic Plan (HSSP IV) 2015-2020[15] and the Tanzania NCD strategic plan 2016-2020.[16] The Tanzania health policy 2007[12] aims to provide guidance about provision of health services for people with NCDs but does not include any advice regarding emergency preparedness actions in relation to NCDs. The Kenya Health Policy[13] provides general guidance on health system response to national disasters, emergencies, and disease outbreaks, however, it does not provide specific guidance in relations to NCD management. The Health Sector Strategic Plan in Tanzania[15] reinforced the importance of integration of NCD clinics into the health care system to enhance accessibility of care but emergency care for NCD was not addressed. The Kenya NCD strategic plan 2015-2020[14] and Tanzania NCD strategic plan 2016-2020[16] addressed NCDs. Both documents promote preventive, curative and rehabilitative services and advocate strengthening leadership and governance, and multi-sectoral collaboration with an emphasis on accountability for the prevention and control of NCDs. However, these strategic plans do not refer to NCD care during emergency situations.

In both countries, participants identified policies, national frameworks, and guidelines for managing NCDs including T2D, which corroborates the findings from the review. KIs also noted that the documents did not cover aspects of care during a pandemic/emergency situation.

> *"Kenya didn't have guidelines that govern diabetes and COVID-19 [pandemics or emergencies] but there were general guidelines for the management of both type two and type one diabetes."* ***Policy Actor_1905_KE***

Modified documents

For peer review only - http://bmjopen.bmj.com/site/about/guidelines.xhtml

There were two documents; one from each country that had been modified during the pandemic to adjust for changing circumstances. In both countries, the NCD strategic plan documents were expiring hence the pandemic situation created an opportunity to highlight emergency situations in the lapsing NCD strategic plans.[26 28] The pre-existing NCD strategic plans[14 16] were modified to include NCD care during the COVID-19 pandemic. KIs confirmed that there were gaps in the previous NCD strategic plans. They described that the COVID-19 pandemic caught them unprepared as there were no plans for continuity of care for NCD patients during this period and this necessitated the modification of the NCD strategic plans during the COVID-19 period.

> *"…Before the onset of COVID-19, I would want to say that we did not have policies or guidelines to manage diabetes [during emergencies] and therefore when COVID-19 came it presented us with some challenge. It was also an eye opener as far as the management of the NCDs is concerned during emergencies or during when we have natural disasters."* ***Policy Actor_1511_KE***

> *"We had the strategic plan for the years 2016 until 2020, which itself was inclusive, so when COVID-19 occurred, we found that since it affects patients living with non-communicable diseases. We should only develop a guideline that will be different from the previous one, because the previous one was on leadership and awareness matters but it [NCD strategic plan 2016-2020] had not described the COVID-19 pandemic"* ***Policy Actor_0002 _TZ***

New documents developed during the pandemic

We identified ten new documents that were developed during the pandemic to manage NCD patients: seven from Kenya and three from Tanzania. These included: one policy (Kenya)[27] and seven guidelines (five in Kenya[18 19 22-24] and two in Tanzania[17 21]) and two other documents which include a government directive in Kenya,[20] and a report in Tanzania.[25] All these documents were created to provide specific guidance on the provision of essential health services and clinical management of NCDs during the pandemic. These documents also provided NCD patients with information on how to manage NCDs during the pandemic.

For peer review only - http://bmjopen.bmj.com/site/about/guidelines.xhtml

For peer review only

Research Question 2: What do the national frameworks, policy models or guidelines include for management of Type 2 Diabetes/NCDs in pandemic/emergency situations?

In both countries, most of the documents reviewed included aspects of national priority setting for managing NCDs in pandemic/emergency situations. The subsequent sub-sections highlight the content of the documents.

Content of the documents

In Kenya, the first interim guidelines for COVID-19 case management were released in April 2020.[18] The guide provided advice on both preventive and clinical management of COVID-19 in Kenya. The interim guidelines were adapted from various international recommendations including from the WHO. An updated version of the guideline was later released in July 2021,[19] and it contains the latest guidelines for the clinical management of COVID-19 in Kenya.

Another interim guideline for essential health services during COVID-19 was released in May 2020.[22] This document was targeted toward healthcare managers and healthcare workers to ensure that people continued to receive essential health services. This was followed by the release of an updated version of the guideline in July 2020.[23] The latter provided guidance for healthcare providers on immediate actions that should be considered to reorganise the health system to ensure continuity of health services during the COVID-19 pandemic for all services including NCD services.

Also in July 2020, another interim guidance on the provision of services from NCDs during the COVID-19 pandemic[24] was released. This document highlighted specific COVID-19 related challenges on NCDs including severity among high-risk persons, COVID-19 actions/responses jeopardising access to care and disrupting lifestyle approaches, disruption of funding and supplies, and the general management of NCDs.

A letter from the Director General of Health in Kenya[20] to the County Executive Committee members for health was also among the first few documents released in mid-April 2020 to request continuity of NCD care amidst the disruption to NCD care provision during the COVID-19 outbreak. This directive instructed NCD clinics to remain operational in all counties during the COVID-19 pandemic.

For peer review only - http://bmjopen.bmj.com/site/about/guidelines.xhtml

In addition, a new Emergency Care Policy[27], distinct from the preceding guidance was issued in July 2021 to provide a roadmap for emergency services to all who need it including those with NCDs. Lastly, in August 2021, the new Kenya National NCD Strategic Plan[28] was launched; it highlighted the disruption to healthcare caused by the COVID-19 pandemic and the need for resilient health systems during health emergencies. It further describes the NCD strategic plan being sensitive to public health emergencies, such as the COVID-19 pandemic but it does not provide guidance on how to better prepare for future emergencies.

In Tanzania, two documents were developed in the early phase of the pandemic. The first was a set of guidelines focused on educating the public on COVID-19 prevention measures, released in April 2020, and it emphasized the need to provide accurate information about the COVID-19 pandemic for patients with NCD.[17] In May 2020, the MOH in Tanzania released another guideline on the provision of NCD and mental health services in the context of the COVID-19 outbreak.[21] This guideline sets out plans for maintaining access to quality health care for NCDs and mental health services during and beyond the pandemic and for strengthening access to COVID-19 pandemic education. It emphasises the importance of protecting people with chronic NCDs, such as T2D patients, from COVID-19 and providing recommendations for doing so, including: hand hygiene, maintaining glycaemic control, staying in touch with healthcare providers, refilling medications before running out and going to health facilities if experiencing COVID-19 symptoms. Additionally, it provided healthcare providers in Tanzania with information on how to manage COVID-19 infection and comorbidities including the major NCDs.

Between July to December 2020 a report on COVID-19 was released in Tanzania to strengthen the capacity of health workers to manage NCDs and COVID-19.[25] In March 2021, the MOH in Tanzania in collaboration with the Tanzania NCD Association (TANCDA) issued a policy brief[26] instead of updating the NCD strategic plan[16] to inform the care aspects of NCDs including diabetes during a pandemic situation. This policy brief reinforced the importance of improving diabetes and NCD management during COVID-19 by empowering NCD patients to take charge of their health, engaging different multi-sectoral groups to support NCDs health services financially, and to promote health awareness.

Access to care and medical supplies

For peer review only - http://bmjopen.bmj.com/site/about/guidelines.xhtml

The documents reviewed described various approaches to care such as modifications to regular practice, workforce issues, how to respond to care disruptions, and health education. In Kenya, several documents were developed during the pandemic to minimise disruptions in care for patients with NCDs due to COVID-19.[22-24] These documents provided mitigation measures such as provision of adequate supply of drugs to patients, patient virtual support (through telemedicine, use of community health volunteers (CHVs) and helplines), counselling, provision of personal protective equipment (PPE) for healthcare providers, and maximum social distancing during facility visits. The Kenya Health Policy[13] provides the long-term development agenda, Vision 2030[29] and global commitments. One of its objectives is to halt and reverse the rising burden of NCD. Access to care and supplies of medicines are discussed but it is not specific to NCDs. The letter by the Director General for Health to all NCD clinics in Kenya noted that access to NCD care was hampered by the closure of the NCD clinics early into the pandemic.[20] This directive addressed the NCD access issue during the pandemic by instructing NCD clinics to remain operational in all counties during the COVID-19 Pandemic. The interim guidance on the provision of services for NCDs during the COVID-19 pandemic[24] provided specific information to ensure care continuity for key NCDs by informing patients of their increased risk of COVID-19, supporting patients to self-manage when appropriate, increasing patients' home supplies of medication and stock needed for their monitoring devices, providing counselling on healthy diets and physical activity, ensuring maximum social distancing during their visits, modifying their routine clinical review frequency and means/mode of delivery. This guideline further provided a section on how to manage diabetes during the COVID-19 pandemic. The document provided advice on strategies people with diabetes can adopt to reduce their risk of COVID-19. This document further acknowledged that there may be instances when the limited healthcare resources could be diverted to care only for those with COVID-19. Some of the documents reviewed encouraged the adoption of virtual consultations and digital diagnostic platforms.[18 23 24 28]

In Tanzania, the guideline[21] and report[25] recognise that the continuity of NCD care was important because of how vulnerable people living with NCDs were during the pandemic. The guideline also highlighted the importance of accessing care to minimise the disruptions in the healthcare system. The guideline emphasized patients' access to essential services during the pandemic and it recommended free provision of all preventive care medical supplies.[21] The report

For peer review only - http://bmjopen.bmj.com/site/about/guidelines.xhtml

also advocated for increased provision of essential medical supplies and access to essential diagnostic equipment and treatment for NCDs during the pandemic.[25]

The KIs in both countries described how access to care was hindered during the pandemic for several reasons including patients with NCDs not wanting to go to health facilities out of fear of contracting the virus and limited skills of healthcare workers to handle NCDs and COVID-19. KIs also confirmed that policies and guidelines were developed to address the access issues experienced during the COVID-19 pandemic.

"You know, most of the health facilities were shut down, generally the whole country was shut down including health facilities and so what remained were just facilities to respond to the COVID. Because of that, several of our clients could not access the services that they required. A letter was sent to the counties by the acting Director General for health to have these clinics open. So that was one of the policies that was issued so that the clients would be able to access the services." ***Policy Actor_1605_KE***

"NCD services were affected in some clinics. Service providers lacked skills, so they didn't know what to do to serve NCD patients. In some facilities, some providers stopped providing services and some clinics were closed, and the patients were afraid to go to the clinics. As a result, it was a challenge on patients' and providers' sides." ***Policy Actor_0002_TZ***

KIs also described that continuity of care for NCD patients was done using alternative strategies. Healthcare providers were encouraged to ensure continuity of care by following up with their NCD patients at the community level either physically with the help of CHVs or virtually.

"…We encouraged the clinicians to do close monitoring and contact tracing with the help of the community volunteers and in facilities which were more advanced, they did it [followed up with patients] virtually." ***Policy Actor_0912_KE***

Monitoring and evaluation tools

There were three documents that provided monitoring and evaluation frameworks; the Kenya Health Policy,[13] the National Strategic Plan for the Prevention and Control of Non-

For peer review only - http://bmjopen.bmj.com/site/about/guidelines.xhtml

Communicable Disease 2021/22 - 2025/26 [28] and the Kenya Emergency Medical Care Policy 2020 – 2030.[27] The Kenya Health Policy includes progress indicators across eight domains and their targets are based on the WHO statistics of the average value of four middle-income countries. One of the targets is to reduce annual deaths due to NCDs by 27% in 2030. Some of the targets included in the National Strategic Plan for the Prevention and Control of NCD 2021/22 - 2025/26[28] are to increase the proportion of funding to NCDs from 48% to 60%, reducing the prevalence of high blood pressure by 25% and reducing the rising burden of diabetes by 10% in 2025. The Kenya Emergency Medical Care Policy 2020 – 2030[27] monitoring and evaluation framework had general targets for emergencies with nothing specific to NCDs. Even though several documents reviewed in Kenya had monitoring & evaluation frameworks, none were focussed on emergency/pandemic care. In Tanzania, none of the documents reviewed mentioned monitoring and evaluation tools.

Research question 3: What are the policy/guideline gaps for the management of Type 2 Diabetes during the COVID-19 pandemic, and what are the opportunities to improve the management of T2D/NCDs?

Gaps identified

Several gaps were identified from the reviewed documents and the key informant interviews from the two countries. These included limited resource allocation and the lack of comprehensiveness of the documents with future preparedness and monitoring and evaluation tools inadequately addressed.

Resource allocation

In both countries, resource allocation for the management of NCDs in pandemic/emergency situations was not described in the guidance/policy documents. KIs noted that this affected the development, dissemination, and implementation of the new policies/guidelines.

KIs stated that the development of the new policies/guidelines was affected by the COVID-19 restrictions on gatherings. In the initial stages of COVID-19, all meetings for the policy/guideline development were moved to virtual platforms. Participants said that the technology needed to access virtual meetings (e.g., data bundles, modems) was under-resourced,

For peer review only

For peer review only - http://bmjopen.bmj.com/site/about/guidelines.xhtml

making participation in the policy/guideline development meetings challenging and strenuous. KIs also suggested that the virtual policy/guideline meetings struggled to reach consensus.

"Yes, there were challenges […] The guideline was done quickly but it was still a tug of war in people's participation: sometimes other people delayed in responding, others did not submit their input, or sometimes others submitted their input after the document has already moved further steps, sometimes a person could participate today or tomorrow, and he does appear [not participate again]. " ***Policy Actor_0003_TZ***

"We had no resources, so we bought our modems. We had to ensure that the [data] bundles were always enough because the meetings were over and over through virtual meetings, so like every other minute you had a virtual meeting. There was no direct resource to the NCD department for COVID-19." ***Policy Actor_1905_KE***

Dissemination of the new policies/guidelines was challenging due to inadequate resources to cover all regions and the COVID-19 restrictions. Participants mentioned that not all the healthcare workers received copies of the new guidelines. Others mentioned that many healthcare workers were not trained on the new guidelines.

"I think the resources were available but probably not adequate. So, for instance, the financial resources to support a full-fledged dissemination across the country...or to support mentorship across all the counties was not adequate." ***Policy Actor_1401_KE***

"About the dissemination, you will remember during the COVID period, the matter of calling people together was restricted, so the dissemination phase was not done properly although I know there was training. For example, TANCDA trained people in Dar es salaam and the Ministry of Health was also doing training in some regions… It was just the distribution of the guideline [was inadequate]. We just shared this document on the online platform" ***Policy Actor_0003_TZ***

For peer review only - http://bmjopen.bmj.com/site/about/guidelines.xhtml

In both countries, the implementation of the new guidelines was a challenge. Participants described the monitoring and evaluation of the new guidelines as a gap due to the limited resources allocated and the restricted movements due to COVID-19. KIs mentioned that they were not aware of how the implementation was progressing.

"So, I may not be able to speak about implementation because I'm not sure what happened after the guidelines were released and shared widely through emails and WhatsApp groups. Whether the healthcare workers used the guidelines or whether they were important or whether they were asking for a revision, or the guidelines were speaking to them, I am not able to say anything about that. I don't know because we had no resources to monitor the implementation." ***Policy Actor_1905_KE***

"…since there was no follow-up done, we were not sure whether the printed 500 copies of the guideline reached the respective health facilities" ***Policy Actor_0002_TZ***

The reallocation of most healthcare funding to address COVID-19 meant there were no funds to cater for the recommendation to give patients with T2D a longer supply of NCD medicines as referred to in reviewed documents in Kenya.[22-24] Interviews with the policy stakeholders in Kenya and Tanzania further confirmed this. In Tanzania specifically, the health insurance coverage was not adjusted to comply with the new guidelines, therefore, the implementation of the recommendation to give patients with T2D a two-to-three-month supply of medications to limit hospital visits was hampered.

"Other support resources are required, [for example,] if someone is to treat a patient … then you need those medicines in place. [However,] the main availability of NCD medicines in the country is half of what is required; it's like 42% out of the targeted 80%.... therefore, there is an inadequate amount to enhance full implementation of these guidelines" ***Policy Actor_1401_KE***

"I remember at one point we even asked the hospitals not to give longer provision of supplies … if for example, you give them three months' supplies it means that you are

For peer review only - http://bmjopen.bmj.com/site/about/guidelines.xhtml

denying someone else, so we decided to go back to the one month supply of medicines and then monitor the situation." ***Policy Actor_1905_KE***

Comprehensiveness of the new policies/guidelines

A gap identified in the review of the documents was the lack of tools needed to monitor and evaluate the progress of the new policy/guidelines. In both countries KIs noted that the guidelines developed lacked monitoring and evaluation tools.

"Sometimes we don't embed [monitoring and evaluation tools] in the supervision [of health facilities], instead, they are included in the supervision tool. Okay, so these guidelines that are disease-specific or aimed at patient management, we don't include them with the M&E framework unless we have a separate framework for supervision." ***Policy Actor_0003_TZ.***

None of the policy/guidelines in Tanzania included plans for managing T2D/NCDs in emergency situations. Participants described this to be a gap even in the new policy/guidelines that were developed during the COVID-19 pandemic.

"Another thing we have come to discover later is that the guideline was still not enough to provide guidance on what to do in an emergency to care for patients with NCDs." ***Policy Actor_0001_TZ.***

Opportunities identified

Participants were able to provide insights into what opportunities they took advantage of to address some of the challenges. KIs in both countries mentioned that the pandemic created an opportunity to establish online platforms to ensure information reached key people within the healthcare sector.

"…in terms of monitoring or supportive supervision that happened maybe not to the extent that we want… but one of the ways we are collecting feedback is [by] empowering the counties and having an NCD focal person… Now we have a common platform both through email [and] we have a WhatsApp group for them where we quickly disseminate any required information..." ***Policy Actor _1004_KE***

For peer review only - http://bmjopen.bmj.com/site/about/guidelines.xhtml

In Tanzania, the COVID-19-pandemic presented an opportunity to use routine supervision meetings as an avenue to disseminate the new policies/guidelines to health facilities. The intention was that the information would be cascaded downwards to all health facility staff.

> *"…but we have also been able to disseminate in the sense of going with the guidelines when we do supervision, this was done in the first group that I was involved in, when we meet the regional health management team (RHMT) we take them through the NCD guideline, but we also provide them with copies for them to distribute in the respective health facilities, we did not go to all council health management team (CHMTs)"* ***Policy Actor_0001_TZ.***

Participants also mentioned the importance of putting forth strategies to strengthen the health system response towards managing NCDs including diabetes in general and during emergency situations. This included prioritising NCDs, multi-sectoral action, multi-level engagements and improved data and monitoring systems to ensure continuity of care.

> *"… [There] should be a multidisciplinary preparedness team which should keep on meeting frequently and if need-be on monthly basis. […] We [also] need to have a very elaborate policy statement as far as management of NCDs during a pandemics is concerned, and this policy should talk of resources strengthening of our systems, monitoring and evaluation, surveillance, and research. [It should also be able to strengthen the capacity of our healthcare providers…. It should also address the issue of medicines and health technologies which are available such that even if the patient does not go to a health facility they can seek service from where one is…"* ***Policy Actor_1511_KE***

Discussion

This study provides a comprehensive review of policies/guidelines that exist to manage NCDs in Kenya and Tanzania during emergency situations with inputs of key NCD stakeholders involved in decision making. This review revealed that prior to the COVID-19 pandemic, both countries did not have comprehensive policies/guidelines that addressed the management of NCDs during pandemic/emergency situations. However, several measures were put in place during the COVID-19 period to address the needs of patients with NCD and T2DM. These measures

For peer review only - http://bmjopen.bmj.com/site/about/guidelines.xhtml

included: updating and developing strategic plans; development of new care guidelines; and release of government directives that guided continuity of care strategies. However, insufficient attention was given to implementation of the new policies and guidelines in both countries. There was in adequate guidance and resources provided to enable effective monitoring and evaluation of progress in relation to disaster preparedness.

The need to develop new and update policies/guidelines in Kenya and Tanzania was similar to other countries.[2] Our study found that the implementation of new policies/guidelines in Kenya and Tanzania faced challenges, for example, inadequate resources hampered the dissemination and training of healthcare workers. There were a lack of key performance targets and a lack of monitoring and evaluation which resulted in poor implementation and reduced effectiveness of the guidelines. A systematic review has revealed that NCD policy development and implementation is often inadequate in Africa.[30] A possible explanation is that healthcare resources are already limited and during the pandemic, these resources were diverted to address COVID-19 infections.[7] Research conducted by the International Federation of Red Cross (IFRC)[31] during the COVID-19 pandemic looking at the role of law and policy in public health emergency preparedness and response found that the pre-existing policies and laws were inadequate to cater to the current COVID-19 pandemic. This was similar to the finding of the study reported here. Due to the rapid development of the new policies/guidelines, a weakness noted was that major components were missed such as monitoring and evaluation and emergency preparedness tools in most of the policies/guidelines.

Some findings were similar across the two countries. For instance, in the initial phase of the COVID-19 pandemic, KIs reported disruption to healthcare services for patients with NCDs in both countries. Therefore, access, availability and utilization of NCD care services were severely affected. The disruptions noted in Kenya and Tanzania were similar to disruptions reported across multiple countries;[2 32] e.g., a WHO report said 75% of countries experienced disruptions to various NCD care services.[2] Supply of NCD medicines was a major issue at multiple levels of the health system,[33] and many people living with NCDs lacked sufficient medicine supplies to manage their conditions, therefore leading to worse health outcomes. There was further evidence to show that LMICs especially countries in SSA were more likely than other countries to

For peer review only - http://bmjopen.bmj.com/site/about/guidelines.xhtml

report disruption of care due to unavailability/stock-outs of medicines and reduced number of healthcare providers at heath facilities[2 34] as was reported by KIs in the current study. The most common reasons reported for not accessing care among people living with NCD in a recent study conducted by Devi et.al were fear of contracting COVID-19 (36.5%), doctors' advice not to go to the health facility (33.7%), and physical inaccessibility due to the lockdowns (27%).[35] This meant that many patients with chronic diseases were not able to receive the care that they needed and resonates with the findings presented here.

The current study shows that both countries had gaps between what was stated in the policy and what was occurring in practice and this was similar to findings from a previous study conducted before COVID-19 looking at the development and implementation process of NCD prevention policies in sub-Saharan Africa.[36] Despite the newly developed or updated policies/guidelines highlighting the need for longer supply of medicines for NCDs, both countries were unable to fully comply with these policies/guidelines due to low medication stocks. In addition, healthcare systems in Tanzania failed to comply with the new policies/guidelines because health insurance schemes were not aligning themselves with the new policies/guidelines.

Strengths and limitations

A major strength in this study was the triangulation of data sources from the desk review with qualitative interviews. Another strength is the use of a comprehensive approach to identify relevant documents and extracting data in a standardised format. Key stakeholders involved in NCD decision making further assisted us in retrieving documents not in the public domain. However, the findings from this study are limited to information from documents that were documented and accessible for review. Another limitation of this review is that we did not include healthcare providers' perspectives, which would have been valuable in understanding the implementation process of the new/updated policies/guidelines as well as the successes and challenges in order to inform recommendations for improvement.

Recommendations

For peer review only - http://bmjopen.bmj.com/site/about/guidelines.xhtml

The COVID-19 pandemic created an opportunity for governments to review and update their national disaster plans. Existing government national disaster plans should integrate NCD management guidelines with national disaster preparedness plans. The plans should also have adequate ring-fenced allocation of resources to ensure proper implementation and monitoring and evaluation of effectiveness of guidelines during emergencies and future pandemics. Governments should strengthen health systems and emergency preparedness by ensuring continuity of service provision for patients with NCDs by offering innovative services such as online consultation platforms, home delivery of diagnostics and ensuring adequate supplies of medication.

Conclusion

By comprehensively analysing the policy response to managing chronic diseases in Kenya and Tanzania, we have identified the effect of the pandemic on continuity of care. Kenya and Tanzania have developed and updated new policies/guidelines to include an emphasis on continuity of care for people with NCDs. Public health emergencies will continue to occur; therefore, there is a strong need to expand these policies and guidelines to cover future emergency situations and to strengthen health system resilience to multiple shocks. This will minimise disruptions in NCD care. Overall, the health systems need to be better prepared to handle disruptions in care by establishing emergency preparedness plans and, adequate resources to implement them.

For peer review only

For peer review only - http://bmjopen.bmj.com/site/about/guidelines.xhtml

Acknowledgements

We would also like to acknowledge the support

Authors' Contribution:

SFM, LK and IM extracted and synthesized data from the policies analysed qualitative data from both countries. SFM and LK jointly conducted the literature review and analysis and wrote the first of the draft manuscript. All the other authors reviewed the draft manuscript, provided input, critical comments and suggested additional analyses. SFM finalized the manuscript which was subsequently approved by all authors.

Funding:

This work was supported by the Medical Research Council and by the National Institute for Health Research (NIHR) [grant number MR/V035924/1] using UK aid from the UK Government to support global health research. The views expressed in this publication are those of the author(s) and not necessarily those of the MRC, NIHR, or the UK government.

Competing interests:

None declared

For peer review only

References

1. Chudasama YV, Gillies CL, Zaccardi F, et al. Impact of COVID-19 on routine care for chronic diseases: a global survey of views from healthcare professionals. *Diabetes & Metabolic Syndrome: Clinical Research & Reviews* 2020;14(5):965-67.
2. World Health Organization - NCD Department. Final Results: Rapid assessment of service delivery for non-communicable diseases (NCDs) during the COVID-19 Pandemic, 2020.
3. Nikoloski Z, Alqunaibet AM, Alfawaz RA, et al. Covid-19 and non-communicable diseases: evidence from a systematic literature review. *BMC public health* 2021;21(1):1-9.
4. Richardson S, Hirsch JS, Narasimhan M, et al. Presenting Characteristics, Comorbidities, and Outcomes Among 5700 Patients Hospitalized With COVID-19 in the New York City Area. *JAMA* 2020;323(20):2052-59. doi: 10.1001/jama.2020.6775
5. Vitiello A, Ferrara F. The impact of COVID-19 in diabetic patient. *Archives of Medicine and Health Sciences* 2020;8(1):167.
6. Schofield J, Leelarathna L, Thabit H. COVID-19: Impact of and on Diabetes. *Diabetes Therapy* 2020;11(7):1429-35.
7. Jordan RE, Adab P, Cheng K. Covid-19: risk factors for severe disease and death: British Medical Journal Publishing Group, 2020.
8. Gilbert M, Pullano G, Pinotti F, et al. Preparedness and vulnerability of African countries against importations of COVID-19: a modelling study. *The Lancet* 2020;395(10227):871-77.
9. Sobngwi E, Ndour-Mbaye M, Boateng KA, et al. Type 2 diabetes control and complications in specialised diabetes care centres of six sub-Saharan African countries: the Diabcare Africa study. *Diabetes research and clinical practice* 2012;95(1):30-36.
10. Gill P, Stewart K, Treasure E, et al. Methods of data collection in qualitative research: interviews and focus groups. *British Dental Journal* 2008;204:291. doi: 10.1038/bdj.2008.192
11. Stemler S. An overview of content analysis. *Practical assessment, research, and evaluation* 2000;7(1):17.
12. Ministry of Health - Tanzania. Tanzania Health Policy (in Kiswahili) 23 Jul 2018 ed, 2007.
13. Kenya. Ministry of Health. Kenya Health Policy 2014-2030: Ministry of Health, 2014.
14. Ministry of Health. The Kenya National Strategy for the Prevention and Control of Non-communicable Diseases - 2015-2020, 2015.
15. Ministry of Health and Social Welfare. Health Sector Strategic Plan (HSSP IV) July 2015 - June 2020, 2015.
16. Government of the Republic of Tanzania, Ministry of Health CD, Gender, Elderly, Children. Strategic and action plan for the prevention and control of non communicable diseases in Tanzania 2016-2020, 2016.
17. Ministry of Health -Community Develeopment G, Elderly and Children,. National Guideline of Clinical Management and Infection Prevention and Control of Novel Coronavirus (COVID-19).
18. Kenyan Ministry of Health (MOH). Interim Guidelines on Management of COVID-19 in Kenya, 2020.
19. Kenya Ministry of Health (MOH). Guidelines on Case Management of COVID-19 in Kenya, 2021.

For peer review only - http://bmjopen.bmj.com/site/about/guidelines.xhtml

20. Ministry of Health - Office of the Director General. Non-Communicable Diseases Clinic During COVID_19 Outbreak, 2020.
21. Ministry of Health CD, Gender, Elderly and Children,. Guidance on the provision of NCD and mental health services in the context of the covid-19 outbreak in Tanzania, 2020.
22. Kenya Ministry of Health (MOH). Interim Guidance on Continuity of Essential Health Services during the Covid-19 Pandemic. 2020
23. Kenya Ministry of Health (MOH). Guidance on Continuity of Essential Health Services during the Covid-19 Pandemic, 2020.
24. Kenya Ministry of Health (MOH). Interim Guidance on Provision of Services for NCDs during the COVID-19 Pandemic, 2020.
25. United Nations. Flash appeal for Covid-19 Tanzania, 2020.
26. Tanzania NCD Alliance (TANCDA). Policy brief on the inclusion of NCDs on universal health coverage in Tanzania, 2021.
27. Kenya Ministry of Health (MOH). Kenya Emergency Medical Care Policy 2020 - 2030, 2021.
28. Department of non-communicable diseases - Ministry of Health. National Strategic Plan for the Prevention and Control of Non-Communicable Disease 2021/22 - 2025/26, 2021.
29. Government of Kenya. Kenya Vision 2030, 2007.
30. Kassa MD, Grace JM. Noncommunicable Diseases Prevention Policies and Their Implementation in Africa: A Systematic Review. *Public Health Reviews* 2022;42:1604310.
31. International Federation of Red Cross (IFRC) Disasater Law. Law and public health emergency preparedness and response: Lessons from the COVID-19 pandemic: International Federation of Red Cross and Red Crescent Societies, 2021:174.
32. Formenti B, Gregori N, Crosato V, et al. The impact of COVID-19 on communicable and non-communicable diseases in Africa: a narrative review. *Le Infezioni in Medicina* 2022;30(1):30.
33. Kiragu ZW, Gathecha G, Mwangi MK, et al. Access to Medicines for Non-Communicable Diseases (NCDS) during COVID-19 in Kenya: A Descriptive Commentary. *Health Systems & Reform* 2021;7(1):e1984865.
34. Owopetu O, Fasehun L-K, Abakporo U. COVID-19: implications for NCDs and the continuity of care in Sub-Saharan Africa. *Global Health Promotion* 2021;28(2):83-86.
35. Devi R, Goodyear-Smith F, Subramaniam K, et al. The impact of COVID-19 on the care of patients with noncommunicable diseases in low-and middle-income countries: an online survey of patient perspectives. *Journal of Patient Experience* 2021;8:23743735211034091.
36. Juma PA, Mohamed SF, Matanje Mwagomba BL, et al. Non-communicable disease prevention policy process in five African countries authors. *BMC Public Health* 2018;18(1):961. doi: 10.1186/s12889-018-5825-7

Figures

Fig 1: Search strategy

For peer review only - http://bmjopen.bmj.com/site/about/guidelines.xhtml

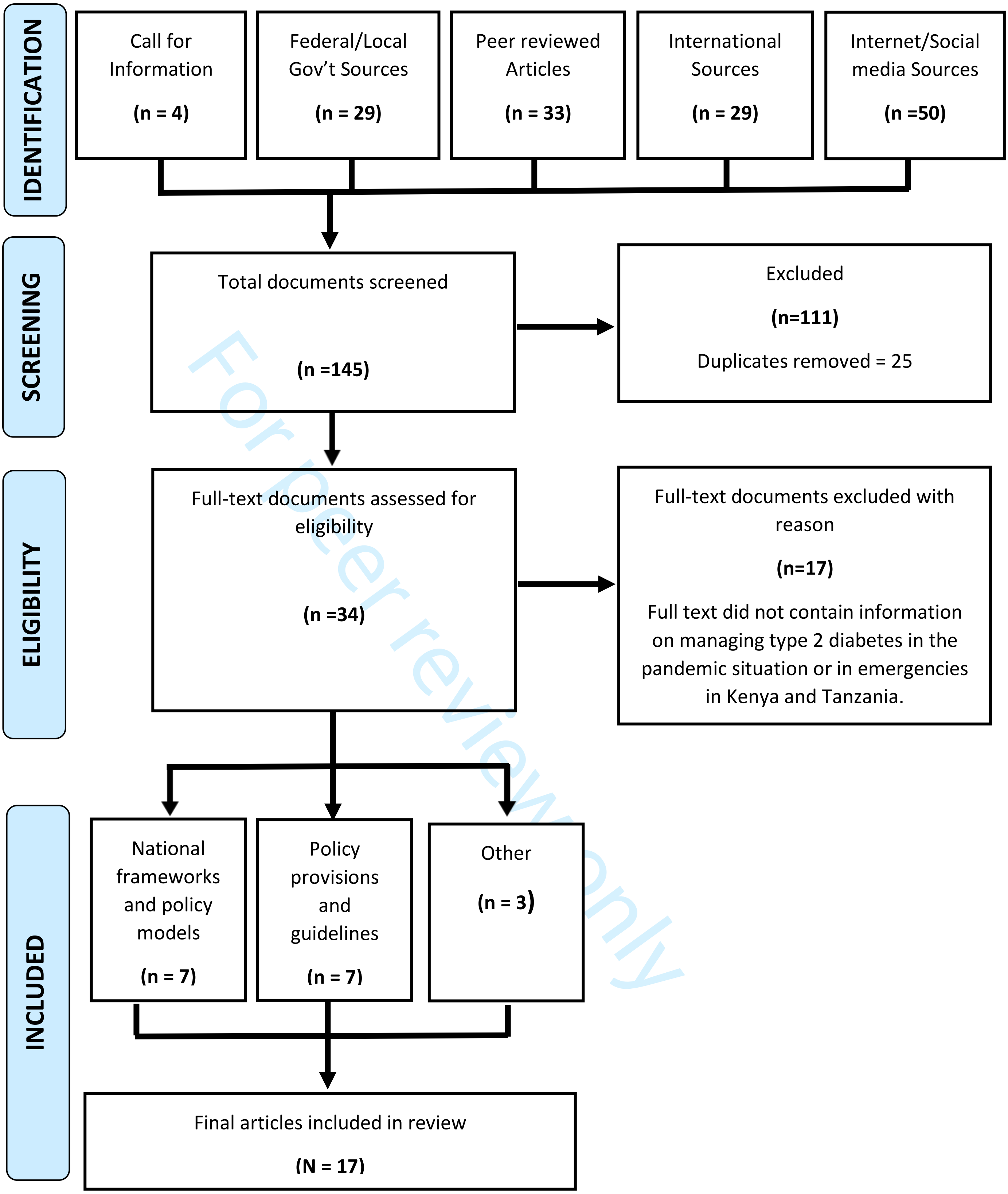

Figure 1: Search strategy

For peer review only

Appendices

Appendix A. Search Criteria for Peer-reviewed literature

1. **Diabetes terms:** diabetes, NCDs, disease management, Type 2 diabetes, diabetes mellitus, chronic disease, non-insulin dependent diabetes, 'type 2 diabetes' OR 'type II diabetes' OR 'diabetes mellitus' OR 'diabetes'
2. **COVID terms**: covid, coronavirus, COVID-19, SARS-COV-2, severe acute respiratory syndrome coronavirus 2, 2019-nCoV
3. **Healthcare:** healthcare, disruption, impact, health services provision, change, continuity of care, medical care
4. **Policies**: policy, guidelines, measurement/assessment/evaluation, strategies, recommendations, plans, impact, research, outcomes, result, approaches, care continuity, SOPs, procedures, frameworks, policy models, laws, bills, tools
5. **Countries**: Kenya, Tanzania

For peer review only

Appendix B: Table 1: Details of search strategy

**Source**	**Details**
Call for information	A call for information was disseminated via email and social media platforms (WhatsApp) to relevant networks/experts including Ministry of Health officials, National NCD department heads, County health departments, diabetes clinic heads, National COVID-19 task force leads; Technical working groups (national or county), Academic researchers who focus on COVID/diabetes care disruption, and NGOs. We first identified a list of potential stakeholders from relevant networks and sent out the call to them. In the call we requested that they share hard or soft copy documents by email or courier related to the management of T2D and other chronic diseases during COVID-19 or other emergency epidemic or pandemic situations.
Peer reviewed literature databases	We searched peer reviewed literature databases of PubMed/Medline, Embase, Google Scholar, Web of Science, OpenGrey using search criteria to identify articles and grey literature that describe diabetes, COVID-19 and health system responses (*see specific search criteria in Appendix A*). Due to time limitations, this was not a comprehensive systematic review, but rather a rapid review of the literature.
Local/ International resources	Since international health agencies such as the World Health Organization have taken the lead both on COVID-19 responses and on management of NCDs, we reviewed documents from international resources, including the United Nations, World Health Organization, and relevant non-governmental agencies. We included WHO country profiles, WHO documents on COVID, and reports from NGOs working in diabetes in Kenya.
Policy documents/drafts from	We reviewed Kenya's health ministry websites including for all documents associated with national NCD strategies, policy documents and committee reports related to T2D and COVID-19, reports from meetings and events, and standard operating procedures. We searched websites including Ministry of health, and any COVID-specific working group, etc. for policies,

For peer review only

relevant national ministries	guidelines, measurement/assessment/evaluation, strategies, recommendations, plans, impact, research, outcomes, result, approaches, care continuity, SOPs, procedures, frameworks, policy models, laws, bills, tools.
Internet search	Given the extensive use of the internet to communicate during the pandemic, we conducted an internet search using google, Google Scholar and other search engines to identify patient support groups, diabetes association's/message boards, newspaper/media articles regarding T2D and COVID.
Social Media	Finally, given the speed with which governments needed to react to COVID-19 developments, many governmental individuals and offices disseminated information to the public on social media. We reviewed twitter pages of the country's Ministry of Health and other high-profile individuals/offices, presidential speeches or other speeches on social media and national NCD groups for information about T2D and COVID-19.

For peer review only

BMJ Open

Policies for type 2 diabetes and non-communicable disease management during the COVID-19 pandemic in Kenya and Tanzania: a desk review and views of decision makers

Journal:	*BMJ Open*
Manuscript ID	bmjopen-2023-073261.R1
Article Type:	Original research
Date Submitted by the Author:	20-Nov-2023
Complete List of Authors:	Mohamed, Shukri; African Population and Health Research Center<br>Kisia, Lyagamula; African Population and Health Research Center<br>Mashiashi, Irene; Ifakara Health Institute<br>Mair, Frances; University of Glasgow, General Practice and Primary Care<br>Wisdom, Jennifer P.; Wisdom Consulting<br>Bunn, Christopher; University of Glasgow College of Social Sciences, Institute of Public Health<br>Gray, Cindy; University of Glasgow, School of Social and Political Sciences; University of Glasgow<br>Kibe , Peter M. ; African Population and Health Research Center<br>Sanya, Richard; African Population and Health Research Center, Health and Wellbeing<br>Karugu, Caroline; African Population and Health Research Center<br>Mtenga, Sally M.; Ifakara Health Institute<br>Binyaruka, Peter; Ifakara Health Institute<br>Asiki, G; African Population and Health Research Center
**Primary Subject Heading**:	Global health
Secondary Subject Heading:	Public health, Global health, Diabetes and endocrinology, Health policy, Cardiovascular medicine
Keywords:	COVID-19, Health policy < HEALTH SERVICES ADMINISTRATION & MANAGEMENT, PUBLIC HEALTH, Chronic Disease, General diabetes < DIABETES & ENDOCRINOLOGY

SCHOLARONE™
Manuscripts

I, the Submitting Author has the right to grant and does grant on behalf of all authors of the Work (as defined in the below author licence), an exclusive licence and/or a non-exclusive licence for contributions from authors who are: i) UK Crown employees; ii) where BMJ has agreed a CC-BY licence shall apply, and/or iii) in accordance with the terms applicable for US Federal Government officers or employees acting as part of their official duties; on a worldwide, perpetual, irrevocable, royalty-free basis to BMJ Publishing Group Ltd ("BMJ") its licensees and where the relevant Journal is co-owned by BMJ to the co-owners of the Journal, to publish the Work in this journal and any other BMJ products and to exploit all rights, as set out in our licence.

The Submitting Author accepts and understands that any supply made under these terms is made by BMJ to the Submitting Author unless you are acting as an employee on behalf of your employer or a postgraduate student of an affiliated institution which is paying any applicable article publishing charge ("APC") for Open Access articles. Where the Submitting Author wishes to make the Work available on an Open Access basis (and intends to pay the relevant APC), the terms of reuse of such Open Access shall be governed by a Creative Commons licence – details of these licences and which Creative Commons licence will apply to this Work are set out in our licence referred to above.

Other than as permitted in any relevant BMJ Author's Self Archiving Policies, I confirm this Work has not been accepted for publication elsewhere, is not being considered for publication elsewhere and does not duplicate material already published. I confirm all authors consent to publication of this Work and authorise the granting of this licence.

For peer review only

Policies for type 2 diabetes and non-communicable disease management during the COVID-19 pandemic in Kenya and Tanzania: a desk review and views of decision makers.

Authors:
Shukri F. Mohamed[1*]
Lyagamula Kisia[1]
Irene Mashiashi[2]
Frances S Mair[3]
Jennifer P. Wisdom[4]
Christopher Bunn[5]
Cindy Gray[5]
Peter Kibe[1]
Richard Sanya[1]
Caroline Karugu[1]
Sally Mtenga[2]
Peter Binyaruka[2]
Gershim Asiki[1]

Author Affiliations
[1] Chronic Disease Management Unit, African Population and Health Research Center (APHRC), Nairobi, Kenya
[2] Ifakara Health Institute, Dar-es-Salaam, Tanzania.
[3] School of Health and Wellbeing, University of Glasgow, UK
[4] Wisdom Consulting, New York, NY, USA
[5] School of Social and Political Sciences, University of Glasgow, UK.

*Corresponding author: Shukri F. Mohamed – smohamed@aphrc.org

Word count - 6161

For peer review only

Abstract

Background: The COVID-19 pandemic caused disruptions in care that adversely affected the management of non-communicable diseases (NCDs) globally. Countries have responded in various ways to support people with NCDs during the pandemic. This study aimed to identify policy gaps, if any, in the management of NCDs, particularly diabetes, during COVID-19 in Kenya and Tanzania to inform recommendations for priority actions for NCD management during any future similar crises.

Methods: We undertook a desk review of pre-existing and newly developed national frameworks, policy models and guidelines for addressing NCDs including type 2 diabetes. This was followed by 13 key informant interviews with stakeholders involved in NCD decision making: six in Kenya; seven in Tanzania. Thematic analysis used to analyse the documents.

Results: Seventeen guidance documents identified (Kenya = 10; Tanzania = 7). These included pre-existing and/or updated policies/strategic plans, guidelines, a letter, a policy brief and a report. Neither country had comprehensive policies/guidelines to ensure continuity of NCD care before the COVID-19 pandemic. However, efforts were made to update pre-existing documents and several more were developed during the pandemic to guide NCD care. Some measures were put in place during the COVID-19 period to ensure continuity of care for patients with NCDs such as longer supply of medicines. Inadequate attention was given to monitoring and evaluation and implementation issues.

Conclusion: Kenya and Tanzania developed and updated some policies/guidelines to include continuity of care in emergencies. However, there were gaps in the documents and between policy/guideline documents and practice. Health systems need to establish disaster preparedness plans that integrate attention to NCD care to enable them to better handle severe disruptions caused by emergencies such as pandemics. Such guidance needs to include contingency planning to enable adequate resources for NCD care and must also address evaluation of implementation effectiveness.

Word count: 297

Key words: Non-communicable diseases, NCDs, diabetes, East Africa, care continuity, care disruption, Sub-Saharan Africa

For peer review only

Strengths and limitations of this study

- Triangulation of data sources from desk review and qualitative interviews.
- Comprehensive approach to identify and extract data in a standardized format.
- Engagement with key stakeholders in NCD decision-making to retrieve non-public documents.
- Findings limited to documented and accessible information.
- The absence of patient and healthcare provider perspectives may have hindered a comprehensive understanding of policy implementation, successes, challenges, to inform recommendations for improvement.

For peer review only

For peer review only - http://bmjopen.bmj.com/site/about/guidelines.xhtml

Introduction

The rapid spread of COVID-19 within a short timeframe resulted in a global pandemic, with a current toll of over six million deaths worldwide as of November 8th, 2023. [1] In Africa, the impact has been significant, with over 9 million confirmed cases and nearly 175,500 deaths reported in the three years since the initial detection of COVID-19.[1] While Africa's share of the pandemic's morbidity and mortality is comparatively lower than other regions, the region faced challenges due to its fragile healthcare system, existing comorbidities, and socio-economic factors.[2-4] The response to the pandemic varied among African countries. For example, Kenya implemented strict lockdown measures [5], while Tanzania opted not to impose such measures.[6] Many countries faced challenges in adequately preparing for the multifaceted challenges posed by COVID-19, putting additional strain on already stretched resources.[7] This necessitated a concerted effort for the development and implementation of measures to mitigate the pandemic's impact and strengthen healthcare systems to address both new and existing health challenges.[8]

The COVID-19 pandemic caused disruptions in care that adversely affected the management of non-communicable diseases (NCDs) globally. Government restrictions implemented in various countries to curb the spread of COVID-19 such as lockdown directives and mobility restrictions impacted management of NCDs by individuals and health systems.[9] A rapid assessment conducted by the World Health Organisation (WHO) across 163 countries revealed that NCD health services including rehabilitation services, hypertension management, and diabetes management, were partially or completely disrupted by the pandemic.[10] These disruptions are likely to have ongoing negative impacts on the management of chronic diseases and progress toward achieving Sustainable Development Goal (SDG) targets. Studies reported that patients with NCDs such as hypertension and diabetes were more likely than those without these comorbidities to be affected by COVID-19 and suffer worse health outcomes if infected.[11, 12]

Despite most countries prioritizing services for four major NCDs (diabetes, hypertension, cancer, and cardiovascular disease) during the COVID-19 pandemic,[10] maintaining routine care for NCDs is a major challenge due to barriers at various levels of healthcare systems. Patients with Type 2 diabetes (T2D) may need continuous and regular blood glucose monitoring and optimal compliance with ongoing drug treatment.[13] Healthcare providers reported that diabetes was the most affected condition by the reduction in healthcare resources.[9] The WHO assessment found

For peer review only - http://bmjopen.bmj.com/site/about/guidelines.xhtml

that 66% of countries surveyed included the continuity of NCD services in the national COVID-19 plans.[10]

Countries have used various strategies to support people with NCDs including diabetes during this pandemic,[14] primarily switching from routine care visits to virtual healthcare visits and communications.[9] Despite these adjustments in service delivery for NCDs, most global healthcare resources focused on the COVID-19 emergency response,[15] particularly in Sub-Saharan Africa (SSA) where healthcare personnel and health resources are inadequate.[16] This resource reallocation disrupted care for patients with NCDs.[17]

In this review, we synthesise policy evidence regarding T2D management during COVID-19 and identified policy gaps, if any, in management of T2D and other NCDs to inform T2D management actions during COVID-19 in Kenya and Tanzania. Our research questions:

(1) Are there pre-existing national frameworks, policy models (priorities, strategies, and directions) and guidelines (instructions) for addressing T2D /NCDs? If so, did they cover pandemic/emergency situations? How have existing frameworks/models (includes formal framework/models and informal statements) been modified for pandemic/emergency situations (e.g., COVID-19) OR how have framework and policy models been created during COVID-19 (including those in development)?

(2) What do the national frameworks, policy models or guidelines tend to include with regards to priority setting (access to care and medical supplies, resource allocation and monitoring and evaluation tools) for management of T2D /NCDs in pandemic/emergency situations?

(3) What are the policy/guideline gaps for the management of T2D during the COVID-19 pandemic (e.g., addressing existing disparities such as socioeconomic status and urban/rural status, addressing health service staff, financial resources for health management, implementation, communication/dissemination), and what are the opportunities to improve the management of T2D/NCDs?

Methods

We conducted a systematic search of policy documents and guiding frameworks on NCDs with a particular focus on T2D in Kenya and Tanzania from October 2021 to March 2022. Our review included pre-existing national frameworks, policy models or guidelines, noting whether these had been modified to include pandemic/emergency situations, as well as new ones developed

For peer review only - http://bmjopen.bmj.com/site/about/guidelines.xhtml

during the COVID-19 pandemic. Identified documents were assessed to ascertain policy/guideline gaps, if any, and opportunities to improve the management of T2D or other NCDs during periods where there are disruptions in care such as the COVID-19 pandemic. While the primary interest was T2D, we also included policy documents which broadly covered NCDs and reviewed their applicability to T2D.

This review was further supplemented by Key Informant Interviews (KIIs)[18] conducted between March and August 2022. The aim of the interviews was to understand how the Kenyan and Tanzanian governments addressed the management of NCDs including T2D during COVID-19, with specific focus on: policy formulation during COVID-19, policy implementation during COVID-19, and gaps noted in the management of NCDs and T2D during COVID-19. Data from the review and the key informant's interview were analyzed separately and then triangulated.

1. <u>Document review</u>

Eligibility criteria

For inclusion in this review documents had to meet four criteria: (1) policy frameworks, policy statements, government statements/pronouncements, publications, emergency preparedness plans, strategies, laws, and operational guidelines for diabetes during COVID-19 (this also included policies under development); (2) specifically mentions NCDs or diabetes or pandemic/emergency or COVID-19; (3) printed in English/Kiswahili; and (4) references Kenya or Tanzania. Documents were excluded from this review if they did not include information on managing type 2 diabetes in a pandemic/emergency situation and if the documents did not reference Kenya or Tanzania.

Search Strategy

A team of seven researchers (Kenya = 4; Tanzania = 3) with backgrounds in medicine, public health, and social sciences conducted the searches and reviewed the documents. All received training on conducting desk reviews. The team coordinator assigned each researcher a different database to search for relevant documentation.

We searched using key text words such as 'Diabetes' AND 'COVID-19', AND 'Healthcare' AND 'Policies' AND 'Kenya' OR 'Tanzania'. Appendix A describes the specific terms used in the search. Using these terms, researchers searched scientific databases

For peer review only - http://bmjopen.bmj.com/site/about/guidelines.xhtml

(PubMed/Medline, Embase, and Web of Science), Ministry of Health (MOH) websites, local/ international resources such as the WHO, and government social media such as the MOH Twitter and Instagram accounts, supplemented with Google searches. We also contacted existing networks and experts in the NCD field through a call for information which was sent via email and WhatsApp groups to ask for any other relevant policy documents. Appendix B provides a detailed explanation of the search strategy for each data source of information.

All retrieved documents were catalogued in an online excel database which facilitated a standardised approach to data extraction from each document. The document characterisation included a unique document number for tracking the title of the document, source of the document (e.g., social media, MOH), and basic information about the document (e.g., year document was produced). The team held weekly "Coding clinics" to ensure consistency in coding of the included documents.

2. <u>Key informant interviews (KII)</u>

Design and participants

The results from the document review were used to inform development of the interview guides for the KIIs. In both Kenya and Tanzania, key informants (KIs) were purposively selected based on their experiences and participation in NCD related policy formulation and implementation processes.

Data collection procedures

A semi-structured interview guide (Appendix C) was used to explore and understand: a) what policies/guidelines/frameworks had been developed during COVID-19; b) who and which sectors had been involved in the policy development and implementation; c), challenges in the formulation and implementation; d) gaps that still exist; and e) recommendations.

Interviews were conducted by two experienced qualitative researchers who were familiar with the NCDs/T2D policy documents. Interviews were conducted in English in Kenya and in Kiswahili in Tanzania. Participants chose between in-person or remote interviews, to ensure convenience and mitigate risk. The interviews lasted approximately 45 minutes. All interviews were audio-recorded and complemented by handwritten notes.

Data management and analysis

For peer review only - http://bmjopen.bmj.com/site/about/guidelines.xhtml

We used content analysis[19] to identify the nature of included documents, the content of the documents, what the documents included regarding priority setting (access to care and medical supplies, resource allocation and monitoring and evaluation tools) and the gaps and opportunities within each document. The information from the desk review was triangulated with the key informants' information to ensure a more in-depth understanding of the synthesised documents and to provide further insights into policy recommendations for NCD management for future emergency situations. The transcripts were coded manually in Word. Two independent researchers, experienced in qualitative data analysis, double coded two transcripts in each country to ensure coding consistency. We obtained a percent agreement of 82%. Disagreements were resolved by rereading the transcripts, discussion and a consensus was reached. In Tanzania, on request from the Tanzania NCD Alliance, a further narrative synthesis of the findings was shared with an NCD expert to identify if there was any important information that was left out, misinterpreted, or missed during the search.

Patient and public involvement

Patients or the public were not involved in the design, or conduct, or reporting, or dissemination of the research. Results of the study were presented to study participants and stakeholders.

Results

Figure 1 describes the search results. After excluding duplicates and records that were not relevant based on title and abstract screening, we identified 34 policy/guidance documents for full text review. After full text review, 17 documents were further excluded because they did not contain information on managing T2D in a pandemic or emergency situation, leaving 17 documents that were included in this review.

Among the included documents, ten were from Kenya and seven were from Tanzania. In Kenya, we found three interim guidelines, two updated guidelines and a letter from the Director General of Health. In Tanzania, two modified guidelines, one policy brief, and one report were included in the review. Each country had one pre-existing national health policy, one pre-existing NCD strategic plan, and an updated NCD strategic plan.

For peer review only - http://bmjopen.bmj.com/site/about/guidelines.xhtml

In total, 13 key informant interviews (Tanzania = 7; Kenya = 6) were conducted with policy actors between March and August 2022. All participants were in the health sector and had different backgrounds in medicine, epidemiology, strategic management, and nutrition. Most of the participants served in various capacities within the MOH. Table 1 provides the characteristics of the KIs interviewed.

Table 1: Characteristics of Key Informants

	**Kenya**	**Tanzania**	**Total**
**Category**			
Government	6	4	**10**
Bilateral organizations/NGOs	0	3	**3**
**Years served in that capacity**			
Less than 1 year	3	0	**3**
1 – 5 years	3	7	**10**
**Total**	**6**	**7**	**13**

The findings are presented by research question.

For peer review only - http://bmjopen.bmj.com/site/about/guidelines.xhtml

Table 2: Description of the documents included in the desk review for Kenya and Tanzania

Type of Document	Name of Document	Country	Dates	Content Description
1. Policy [20]	Tanzania Health Policy 2007	Tanzania	2007	Guides development of NCD guidelines, emphasizing health service provision, equity, capacity building, and awareness on NCD management and prevention.
2. Policy [21]	Kenya Health Policy 2014-2030	Kenya	2014	Provides overall direction for significant health improvement, aligning with Kenya's long-term development agenda and global commitments to address the rising burden of NCDs.
3. National Framework [22]	National Strategy for the Prevention and Control of NCDs 2015 -2020	Kenya	2015 - 2020	Emphasizes an integrated approach to reduce the burden of NCDs, providing evidence-based interventions for sustainable socioeconomic development.
4. National Framework [23]	Health Sector Strategic Plan (HSSP IV)	Tanzania	2015-2020	Advocates for integrating NCD clinics into the healthcare system, emphasizing access to care, NCD prevention, and early detection.
5. National framework [24]	Strategic and action plan for the prevention and control of NCDs in Tanzania 2016 – 2020	Tanzania	2016-2020	Advocates for NCD prevention and control at the national level, emphasizing leadership, governance, multisectoral collaboration, and accountability.
6. Guideline [25]	National guideline of clinical management and infection prevention and control of novel coronavirus (COVID-19)	Tanzania	April 2020	Educates on COVID-19 preventive measures, emphasizing accurate information provision for people with NCDs.
7. Guideline [26]<br>8. Guideline [27]	Interim Guidelines on Management of COVID-19 in Kenya<br>Updated version: Guidelines on Case Management of COVID-19 in Kenya	Kenya	April 2020<br>July, 2021	Combines preventive and clinical management guidelines for COVID-19, updated version includes the latest clinical management guidelines.

For peer review only - http://bmjopen.bmj.com/site/about/guidelines.xhtml
For peer review only

9. Letter [28]	Non-Communicable Diseases Clinic During COVID-19 Outbreak	Kenya	Mid-April 2020	National directive for NCD clinics to remain operational during the COVID-19 pandemic.
10. Guideline [29]	Guidance on the provision of NCD and mental health services in the context of the COVID-19 outbreak in Tanzania	Tanzania	May 2020	Targets strengthening NCD service provision during COVID-19, including capacity building, health promotion, facility restructuring, and continuity of essential services.
11. Guideline [30]<br><br>12. Guidelines [31]	Interim Guidance on Continuity of Essential Health Services during the Covid-19 Pandemic.<br><br>Updated version:<br>Guidance on Continuity of Essential Health Services during the COVID-19 Pandemic	Kenya	May 2020<br><br>July 2020	Provides guidance for reorganizing the health system to ensure continuity of health services during the COVID-19 pandemic, including NCD services.
13. Guideline [32]	Interim Guidance on Provision of Services for NCDs during the COVID-19 Pandemic	Kenya	July 2020	Highlights COVID-19-related challenges on NCDs and their management.
14. Report [33]	Flash appeal for COVID-19 Tanzania	Tanzania	July–Dec 2020	Discusses capacity building for health workers, mentorship on NCD and COVID-19 management, information dissemination, and health promotion strategies.
15. Policy Brief [34]	Tanzania NCD Alliance (TANCDA) policy brief on the inclusion of NCDs on universal health coverage in Tanzania	Tanzania	March 2021	Recommends actions for quality NCD care, health financing, multisectoral collaboration, and health awareness during the COVID-19 pandemic.
16. Policy [35]	Kenya Emergency Medical Care Policy 2020 – 2030	Kenya	July 2021	Provides guidance, structures, resources, regulations, and standards for establishing an emergency medical care system, addressing emergency conditions caused by NCDs.
17. National framework [36]	National Strategic Plan for the Prevention and Control of Non-Communicable Disease 2021/22 - 2025/26	Kenya	August 2021	Provides direction on sectoral and multi-sectoral coordination, leadership, and governance for NCD prevention, research promotion, and surveillance enhancement.

For peer review only - http://bmjopen.bmj.com/site/about/guidelines.xhtml

Research Question 1: What national frameworks, policy models and guidelines for addressing Type 2 Diabetes/NCDs pre-existed and which ones were modified or developed during the COVID-19 pandemic?

Pre-existing documents

There were five documents in Kenya and Tanzania that addressed NCDs or emergency situations prior to the COVID-19 pandemic but none specifically provided guidance on NCD preparedness in an emergency situation. Table 2 and Appendix D provides a brief description of the included documents. The pre-existing documents include: the Tanzania health policy 2007,[20] the Kenya health policy 2014,[21] the Kenya NCD strategic plan 2015-2020,[22] the Tanzania Health Sector Strategic Plan (HSSP IV) 2015-2020[23] and the Tanzania NCD strategic plan 2016-2020.[24] The Tanzania health policy 2007[20] aims to provide guidance about provision of health services for people with NCDs but does not include any advice regarding emergency preparedness actions in relation to NCDs. The Kenya Health Policy[21] provides general guidance on health system response to national disasters, emergencies, and disease outbreaks, however, it does not provide specific guidance in relations to NCD management. The Health Sector Strategic Plan in Tanzania[23] reinforced the importance of integration of NCD clinics into the health care system to enhance accessibility of care but emergency care for NCD was not addressed. The Kenya NCD strategic plan 2015-2020[22] and Tanzania NCD strategic plan 2016-2020[24] addressed NCDs. Both documents promote preventive, curative and rehabilitative services and advocate strengthening leadership and governance, and multi-sectoral collaboration with an emphasis on accountability for the prevention and control of NCDs. However, these strategic plans do not refer to NCD care during emergency situations.

In both countries, participants identified policies, national frameworks, and guidelines for managing NCDs including T2D, which corroborates the findings from the review. KIs also noted that the documents did not cover aspects of care during a pandemic/emergency situation.

> *"Kenya didn't have guidelines that govern diabetes and COVID-19 [pandemics or emergencies] but there were general guidelines for the management of both type two and type one diabetes."* ***Policy Actor_1905_KE***

Modified documents

For peer review only - http://bmjopen.bmj.com/site/about/guidelines.xhtml

There were two documents; one from each country that had been modified during the pandemic to adjust for changing circumstances. In both countries, the NCD strategic plan documents were expiring hence the pandemic situation created an opportunity to highlight emergency situations in the lapsing NCD strategic plans.[34, 36] The pre-existing NCD strategic plans[22, 24] were modified to include NCD care during the COVID-19 pandemic. KIs confirmed that there were gaps in the previous NCD strategic plans. They described that the COVID-19 pandemic caught them unprepared as there were no plans for continuity of care for NCD patients during this period and this necessitated the modification of the NCD strategic plans during the COVID-19 period.

> *"...Before the onset of COVID-19, I would want to say that we did not have policies or guidelines to manage diabetes [during emergencies] and therefore when COVID-19 came it presented us with some challenge. It was also an eye opener as far as the management of the NCDs is concerned during emergencies or during when we have natural disasters."* ***Policy Actor_1511_KE***

> *"We had the strategic plan for the years 2016 until 2020, which itself was inclusive, so when COVID-19 occurred, we found that since it affects patients living with non-communicable diseases. We should only develop a guideline that will be different from the previous one, because the previous one was on leadership and awareness matters but it [NCD strategic plan 2016-2020] had not described the COVID-19 pandemic"* ***Policy Actor_0002 _TZ***

New documents developed during the pandemic

We identified ten new documents that were developed during the pandemic to manage NCD patients: seven from Kenya and three from Tanzania. These included: one policy (Kenya)[35] and seven guidelines (five in Kenya[26, 27, 30-32] and two in Tanzania[25, 29]) and two other documents which include a government directive in Kenya,[28] and a report in Tanzania.[33] All these documents were created to provide specific guidance on the provision of essential health services and clinical management of NCDs during the pandemic. These documents also provided NCD patients with information on how to manage NCDs during the pandemic.

For peer review only - http://bmjopen.bmj.com/site/about/guidelines.xhtml

Research Question 2: What do the national frameworks, policy models or guidelines include for management of Type 2 Diabetes/NCDs in pandemic/emergency situations?

In both countries, most of the documents reviewed included aspects of national priority setting for managing NCDs in pandemic/emergency situations. The subsequent sub-sections highlight the content of the documents.

Content of the documents

In Kenya, the first interim guidelines for COVID-19 case management were released in April 2020.[26] The guide provided advice on both preventive and clinical management of COVID-19 in Kenya. The interim guidelines were adapted from various international recommendations including from the WHO. An updated version of the guideline was later released in July 2021,[27] and it contains the latest guidelines for the clinical management of COVID-19 in Kenya.

Another interim guideline for essential health services during COVID-19 was released in May 2020.[30] This document was targeted toward healthcare managers and healthcare workers to ensure that people continued to receive essential health services. This was followed by the release of an updated version of the guideline in July 2020.[31] The latter provided guidance for healthcare providers on immediate actions that should be considered to reorganise the health system to ensure continuity of health services during the COVID-19 pandemic for all services including NCD services.

Also in July 2020, another interim guidance on the provision of services from NCDs during the COVID-19 pandemic[32] was released. This document highlighted specific COVID-19 related challenges on NCDs including severity among high-risk persons, COVID-19 actions/responses jeopardising access to care and disrupting lifestyle approaches, disruption of funding and supplies, and the general management of NCDs.

A letter from the Director General of Health in Kenya[28] to the County Executive Committee members for health was also among the first few documents released in mid-April 2020 to request continuity of NCD care amidst the disruption to NCD care provision during the COVID-19 outbreak. This directive instructed NCD clinics to remain operational in all counties during the COVID-19 pandemic.

For peer review only - http://bmjopen.bmj.com/site/about/guidelines.xhtml

In addition, a new Emergency Care Policy[35], distinct from the preceding guidance was issued in July 2021 to provide a roadmap for emergency services to all who need it including those with NCDs. Lastly, in August 2021, the new Kenya National NCD Strategic Plan[36] was launched; it highlighted the disruption to healthcare caused by the COVID-19 pandemic and the need for resilient health systems during health emergencies. It further describes the NCD strategic plan being sensitive to public health emergencies, such as the COVID-19 pandemic but it does not provide guidance on how to better prepare for future emergencies.

In Tanzania, two documents were developed in the early phase of the pandemic. The first was a set of guidelines focused on educating the public on COVID-19 prevention measures, released in April 2020, and it emphasized the need to provide accurate information about the COVID-19 pandemic for patients with NCD.[25] In May 2020, the MOH in Tanzania released another guideline on the provision of NCD and mental health services in the context of the COVID-19 outbreak.[29] This guideline sets out plans for maintaining access to quality health care for NCDs and mental health services during and beyond the pandemic and for strengthening access to COVID-19 pandemic education. It emphasises the importance of protecting people with chronic NCDs, such as T2D patients, from COVID-19 and providing recommendations for doing so, including: hand hygiene, maintaining glycaemic control, staying in touch with healthcare providers, refilling medications before running out and going to health facilities if experiencing COVID-19 symptoms. Additionally, it provided healthcare providers in Tanzania with information on how to manage COVID-19 infection and comorbidities including the major NCDs.

Between July to December 2020 a report on COVID-19 was released in Tanzania to strengthen the capacity of health workers to manage NCDs and COVID-19.[33] In March 2021, the MOH in Tanzania in collaboration with the Tanzania NCD Association (TANCDA) issued a policy brief[34] instead of updating the NCD strategic plan[24] to inform the care aspects of NCDs including diabetes during a pandemic situation. This policy brief reinforced the importance of improving diabetes and NCD management during COVID-19 by empowering NCD patients to take charge of their health, engaging different multi-sectoral groups to support NCDs health services financially, and to promote health awareness.

Access to care and medical supplies

For peer review only - http://bmjopen.bmj.com/site/about/guidelines.xhtml

The documents reviewed described various approaches to care such as modifications to regular practice, workforce issues, how to respond to care disruptions, and health education. In Kenya, several documents were developed during the pandemic to minimise disruptions in care for patients with NCDs due to COVID-19.[30-32] These documents provided mitigation measures such as provision of adequate supply of drugs to patients, patient virtual support (through telemedicine, use of community health volunteers (CHVs) and helplines), counselling, provision of personal protective equipment (PPE) for healthcare providers, and maximum social distancing during facility visits. The Kenya Health Policy[21] provides the long-term development agenda, Vision 2030[37] and global commitments. One of its objectives is to halt and reverse the rising burden of NCD. Access to care and supplies of medicines are discussed but it is not specific to NCDs. The letter by the Director General for Health to all NCD clinics in Kenya noted that access to NCD care was hampered by the closure of the NCD clinics early into the pandemic.[28] This directive addressed the NCD access issue during the pandemic by instructing NCD clinics to remain operational in all counties during the COVID-19 Pandemic. The interim guidance on the provision of services for NCDs during the COVID-19 pandemic[32] provided specific information to ensure care continuity for key NCDs by informing patients of their increased risk of COVID-19, supporting patients to self-manage when appropriate, increasing patients' home supplies of medication and stock needed for their monitoring devices, providing counselling on healthy diets and physical activity, ensuring maximum social distancing during their visits, modifying their routine clinical review frequency and means/mode of delivery. This guideline further provided a section on how to manage diabetes during the COVID-19 pandemic. The document provided advice on strategies people with diabetes can adopt to reduce their risk of COVID-19. This document further acknowledged that there may be instances when the limited healthcare resources could be diverted to care only for those with COVID-19. Some of the documents reviewed encouraged the adoption of virtual consultations and digital diagnostic platforms.[26, 31, 32, 36]

In Tanzania, the guideline[29] and report[33] recognise that the continuity of NCD care was important because of how vulnerable people living with NCDs were during the pandemic. The guideline also highlighted the importance of accessing care to minimise the disruptions in the healthcare system. The guideline emphasized patients' access to essential services during the pandemic and it recommended free provision of all preventive care medical supplies.[29] The

For peer review only - http://bmjopen.bmj.com/site/about/guidelines.xhtml

report also advocated for increased provision of essential medical supplies and access to essential diagnostic equipment and treatment for NCDs during the pandemic.[33]

The KIs in both countries described how access to care was hindered during the pandemic for several reasons including patients with NCDs not wanting to go to health facilities out of fear of contracting the virus and limited skills of healthcare workers to handle NCDs and COVID-19. KIs also confirmed that policies and guidelines were developed to address the access issues experienced during the COVID-19 pandemic.

"You know, most of the health facilities were shut down, generally the whole country was shut down including health facilities and so what remained were just facilities to respond to the COVID. Because of that, several of our clients could not access the services that they required. A letter was sent to the counties by the acting Director General for health to have these clinics open. So that was one of the policies that was issued so that the clients would be able to access the services." ***Policy Actor_1605_KE***

"NCD services were affected in some clinics. Service providers lacked skills, so they didn't know what to do to serve NCD patients. In some facilities, some providers stopped providing services and some clinics were closed, and the patients were afraid to go to the clinics. As a result, it was a challenge on patients' and providers' sides." ***Policy Actor_0002_TZ***

KIs also described that continuity of care for NCD patients was done using alternative strategies. Healthcare providers were encouraged to ensure continuity of care by following up with their NCD patients at the community level either physically with the help of CHVs or virtually.

"…We encouraged the clinicians to do close monitoring and contact tracing with the help of the community volunteers and in facilities which were more advanced, they did it [followed up with patients] virtually." ***Policy Actor_0912_KE***

Monitoring and evaluation tools

There were three documents that provided monitoring and evaluation frameworks; the Kenya Health Policy,[21] the National Strategic Plan for the Prevention and Control of Non-

For peer review only - http://bmjopen.bmj.com/site/about/guidelines.xhtml

Communicable Disease 2021/22 - 2025/26 [36] and the Kenya Emergency Medical Care Policy 2020 – 2030.[35] The Kenya Health Policy includes progress indicators across eight domains and their targets are based on the WHO statistics of the average value of four middle-income countries. One of the targets is to reduce annual deaths due to NCDs by 27% in 2030. Some of the targets included in the National Strategic Plan for the Prevention and Control of NCD 2021/22 - 2025/26[36] are to increase the proportion of funding to NCDs from 48% to 60%, reducing the prevalence of high blood pressure by 25% and reducing the rising burden of diabetes by 10% in 2025. The Kenya Emergency Medical Care Policy 2020 – 2030[35] monitoring and evaluation framework had general targets for emergencies with nothing specific to NCDs. Even though several documents reviewed in Kenya had monitoring & evaluation frameworks, none were focussed on emergency/pandemic care. In Tanzania, none of the documents reviewed mentioned monitoring and evaluation tools.

Research question 3: What are the policy/guideline gaps for the management of Type 2 Diabetes during the COVID-19 pandemic, and what are the opportunities to improve the management of T2D/NCDs?

Gaps identified

Several gaps were identified from the reviewed documents and the key informant interviews from the two countries. These included limited resource allocation and the lack of comprehensiveness of the documents with future preparedness and monitoring and evaluation tools inadequately addressed.

Resource allocation

In both countries, resource allocation for the management of NCDs in pandemic/emergency situations was not described in the guidance/policy documents. KIs noted that this affected the development, dissemination, and implementation of the new policies/guidelines.

KIs stated that the development of the new policies/guidelines was affected by the COVID-19 restrictions on gatherings. In the initial stages of COVID-19, all meetings for the policy/guideline development were moved to virtual platforms. Participants said that the technology needed to access virtual meetings (e.g., data bundles, modems) was under-resourced,

For peer review only - http://bmjopen.bmj.com/site/about/guidelines.xhtml

making participation in the policy/guideline development meetings challenging and strenuous. KIs also suggested that the virtual policy/guideline meetings struggled to reach consensus.

"Yes, there were challenges […] The guideline was done quickly but it was still a tug of war in people's participation: sometimes other people delayed in responding, others did not submit their input, or sometimes others submitted their input after the document has already moved further steps, sometimes a person could participate today or tomorrow, and he does appear [not participate again]. " ***Policy Actor_0003_TZ***

"We had no resources, so we bought our modems. We had to ensure that the [data] bundles were always enough because the meetings were over and over through virtual meetings, so like every other minute you had a virtual meeting. There was no direct resource to the NCD department for COVID-19." ***Policy Actor_1905_KE***

Dissemination of the new policies/guidelines was challenging due to inadequate resources to cover all regions and the COVID-19 restrictions. Participants mentioned that not all the healthcare workers received copies of the new guidelines. Others mentioned that many healthcare workers were not trained on the new guidelines.

"I think the resources were available but probably not adequate. So, for instance, the financial resources to support a full-fledged dissemination across the country...or to support mentorship across all the counties was not adequate." ***Policy Actor_1401_KE***

"About the dissemination, you will remember during the COVID period, the matter of calling people together was restricted, so the dissemination phase was not done properly although I know there was training. For example, TANCDA trained people in Dar es salaam and the Ministry of Health was also doing training in some regions… It was just the distribution of the guideline [was inadequate]. We just shared this document on the online platform" ***Policy Actor_0003_TZ***

For peer review only - http://bmjopen.bmj.com/site/about/guidelines.xhtml

In both countries, the implementation of the new guidelines was a challenge. Participants described the monitoring and evaluation of the new guidelines as a gap due to the limited resources allocated and the restricted movements due to COVID-19. KIs mentioned that they were not aware of how the implementation was progressing.

"So, I may not be able to speak about implementation because I'm not sure what happened after the guidelines were released and shared widely through emails and WhatsApp groups. Whether the healthcare workers used the guidelines or whether they were important or whether they were asking for a revision, or the guidelines were speaking to them, I am not able to say anything about that. I don't know because we had no resources to monitor the implementation." ***Policy Actor_1905_KE***

"…since there was no follow-up done, we were not sure whether the printed 500 copies of the guideline reached the respective health facilities" ***Policy Actor_0002_TZ***

The reallocation of most healthcare funding to address COVID-19 meant there were no funds to cater for the recommendation to give patients with T2D a longer supply of NCD medicines as referred to in reviewed documents in Kenya.[30-32] Interviews with the policy stakeholders in Kenya and Tanzania further confirmed this. In Tanzania specifically, the health insurance coverage was not adjusted to comply with the new guidelines, therefore, the implementation of the recommendation to give patients with T2D a two-to-three-month supply of medications to limit hospital visits was hampered.

"Other support resources are required, [for example,] if someone is to treat a patient … then you need those medicines in place. [However,] the main availability of NCD medicines in the country is half of what is required; it's like 42% out of the targeted 80%.... therefore, there is an inadequate amount to enhance full implementation of these guidelines" ***Policy Actor_1401_KE***

"I remember at one point we even asked the hospitals not to give longer provision of supplies … if for example, you give them three months' supplies it means that you are

For peer review only - http://bmjopen.bmj.com/site/about/guidelines.xhtml

denying someone else, so we decided to go back to the one month supply of medicines and then monitor the situation." ***Policy Actor_1905_KE***

Comprehensiveness of the new policies/guidelines

A gap identified in the review of the documents was the lack of tools needed to monitor and evaluate the progress of the new policy/guidelines. In both countries KIs noted that the guidelines developed lacked monitoring and evaluation tools.

"Sometimes we don't embed [monitoring and evaluation tools] in the supervision [of health facilities], instead, they are included in the supervision tool. Okay, so these guidelines that are disease-specific or aimed at patient management, we don't include them with the M&E framework unless we have a separate framework for supervision." ***Policy Actor_0003_TZ.***

None of the policy/guidelines in Tanzania included plans for managing T2D/NCDs in emergency situations. Participants described this to be a gap even in the new policy/guidelines that were developed during the COVID-19 pandemic.

"Another thing we have come to discover later is that the guideline was still not enough to provide guidance on what to do in an emergency to care for patients with NCDs." ***Policy Actor_0001_TZ.***

Opportunities identified

Participants were able to provide insights into what opportunities they took advantage of to address some of the challenges. KIs in both countries mentioned that the pandemic created an opportunity to establish online platforms to ensure information reached key people within the healthcare sector.

"...in terms of monitoring or supportive supervision that happened maybe not to the extent that we want… but one of the ways we are collecting feedback is [by] empowering the counties and having an NCD focal person… Now we have a common platform both through email [and] we have a WhatsApp group for them where we quickly disseminate any required information..." ***Policy Actor _1004_KE***

For peer review only - http://bmjopen.bmj.com/site/about/guidelines.xhtml

In Tanzania, the COVID-19-pandemic presented an opportunity to use routine supervision meetings as an avenue to disseminate the new policies/guidelines to health facilities. The intention was that the information would be cascaded downwards to all health facility staff.

"…but we have also been able to disseminate in the sense of going with the guidelines when we do supervision, this was done in the first group that I was involved in, when we meet the regional health management team (RHMT) we take them through the NCD guideline, but we also provide them with copies for them to distribute in the respective health facilities, we did not go to all council health management team (CHMTs)" ***Policy Actor_0001_TZ.***

Participants also mentioned the importance of putting forth strategies to strengthen the health system response towards managing NCDs including diabetes in general and during emergency situations. This included prioritising NCDs, multi-sectoral action, multi-level engagements and improved data and monitoring systems to ensure continuity of care.

"… [There] should be a multidisciplinary preparedness team which should keep on meeting frequently and if need-be on monthly basis. […] We [also] need to have a very elaborate policy statement as far as management of NCDs during a pandemics is concerned, and this policy should talk of resources strengthening of our systems, monitoring and evaluation, surveillance, and research. [It should also be able to strengthen the capacity of our healthcare providers.… It should also address the issue of medicines and health technologies which are available such that even if the patient does not go to a health facility they can seek service from where one is…" ***Policy Actor_1511_KE***

Discussion

This study provides a comprehensive review of policies/guidelines that exist to manage NCDs in Kenya and Tanzania during emergency situations with inputs of key NCD stakeholders involved in decision making. This review revealed that prior to the COVID-19 pandemic, both countries did not have comprehensive policies/guidelines that addressed the management of NCDs during pandemic/emergency situations. However, several measures were put in place during the COVID-19 period to address the needs of patients with NCD and T2DM. These measures

For peer review only - http://bmjopen.bmj.com/site/about/guidelines.xhtml

included: updating and developing strategic plans; development of new care guidelines; and release of government directives that guided continuity of care strategies. However, insufficient attention was given to implementation of the new policies and guidelines in both countries. There was inadequate guidance and resources provided to enable effective monitoring and evaluation of progress in relation to disaster preparedness.

The need to develop new and update policies/guidelines in Kenya and Tanzania was similar to other countries.[10] Our study found that the implementation of new policies/guidelines in Kenya and Tanzania faced challenges, for example, inadequate resources hampered the dissemination and training of healthcare workers. There were a lack of key performance targets and a lack of monitoring and evaluation which resulted in poor implementation and reduced effectiveness of the guidelines. A systematic review has revealed that NCD policy development and implementation is often inadequate in Africa.[38] A possible explanation is that healthcare resources are already limited and during the pandemic, these resources were diverted to address COVID-19 infections.[15] Research conducted by the International Federation of Red Cross (IFRC)[39] during the COVID-19 pandemic looking at the role of law and policy in public health emergency preparedness and response found that the pre-existing policies and laws were inadequate to cater to the current COVID-19 pandemic. This was similar to the finding of the study reported here. Due to the rapid development of the new policies/guidelines, a weakness noted was that major components were missed such as monitoring and evaluation and emergency preparedness tools in most of the policies/guidelines.

Some findings were similar across the two countries. For instance, in the initial phase of the COVID-19 pandemic, KIs reported disruption to healthcare services for patients with NCDs in both countries. Therefore, access, availability and utilization of NCD care services were severely affected. The disruptions noted in Kenya and Tanzania were similar to disruptions reported across multiple countries;[3, 10] e.g., a WHO report said 75% of countries experienced disruptions to various NCD care services.[10] Supply of NCD medicines was a major issue at multiple levels of the health system,[40] and many people living with NCDs lacked sufficient medicine supplies to manage their conditions, therefore leading to worse health outcomes. There was further evidence to show that LMICs especially countries in SSA were more likely than other

For peer review only - http://bmjopen.bmj.com/site/about/guidelines.xhtml

countries to report disruption of care due to unavailability/stock-outs of medicines and reduced number of healthcare providers at heath facilities[10, 41] as was reported by KIs in the current study. The most common reasons reported for not accessing care among people living with NCD in a recent study conducted by Devi et.al were fear of contracting COVID-19 (36.5%), doctors' advice not to go to the health facility (33.7%), and physical inaccessibility due to the lockdowns (27%).[42] This meant that many patients with chronic diseases were not able to receive the care that they needed and resonates with the findings presented here.

The current study shows that both countries had gaps between what was stated in the policy and what was occurring in practice and this was similar to findings from a previous study conducted before COVID-19 looking at the development and implementation process of NCD prevention policies in sub-Saharan Africa.[43] Despite the newly developed or updated policies/guidelines highlighting the need for longer supply of medicines for NCDs, both countries were unable to fully comply with these policies/guidelines due to low medication stocks. In addition, healthcare systems in Tanzania failed to comply with the new policies/guidelines because health insurance schemes were not aligning themselves with the new policies/guidelines.

Strengths and limitations

A major strength in this study was the triangulation of data sources from the desk review with qualitative interviews. Another strength is the use of a comprehensive approach to identify relevant documents and extracting data in a standardised format. Key stakeholders involved in NCD decision making further assisted us in retrieving documents not in the public domain. However, the findings from this study are limited to information from documents that were documented and accessible for review. Another limitation of this review is that we did not include patients 'and healthcare providers' perspectives, which would have been valuable in understanding the implementation process of the new/updated policies/guidelines as well as the successes and challenges in order to inform recommendations for improvement.

Recommendations

For peer review only - http://bmjopen.bmj.com/site/about/guidelines.xhtml

The COVID-19 pandemic created an opportunity for governments to review and update their national disaster plans. Existing government national disaster plans should integrate NCD management guidelines with national disaster preparedness plans. The plans should also have adequate ring-fenced allocation of resources to ensure proper implementation and monitoring and evaluation of effectiveness of guidelines during emergencies and future pandemics. Governments should strengthen health systems and emergency preparedness by ensuring continuity of service provision for patients with NCDs by offering innovative services such as online consultation platforms, home delivery of diagnostics and ensuring adequate supplies of medication. Additional research on this topic has the potential to offer valuable insights on future developments.

Conclusion

By comprehensively analysing the policy response to managing chronic diseases in Kenya and Tanzania, we have identified the effect of the pandemic on continuity of care. Kenya and Tanzania have developed and updated new policies/guidelines to include an emphasis on continuity of care for people with NCDs. There is a pressing need to develop disaster/emergency preparedness plans that encompass NCD care and allocate sufficient resources to ensure their effective implementation. This is crucial for effectively managing future emergency situations while strengthening the resilience of health systems to withstand multiple shocks. There is also need to promote and build capacities for implementation research in Kenya and Tanzania.

For peer review only

Acknowledgements

We thank the field staff in both countries for their dedication and commitment. We also extend our thanks to the experts who generously shared their time, knowledge, and experiences, which were fundamental to the success of this study.

Authors' Contribution:

SFM, LK and IM extracted and synthesized data from the policies and analysed the qualitative data from both countries. SFM and LK jointly conducted the literature review and analysis and wrote the first draft manuscript. All the other authors (IM, FSM, JPW, CB, CG, PK, RS, CK, SM, PB & GA) reviewed the draft manuscript, provided input, provided critical comments and suggested additional analyses. All authors contributed substantially to the development, revision, and finalisation of the manuscript. SFM finalized the manuscript, which was subsequently approved by all authors.

Funding:

This work was supported by the Medical Research Council and by the National Institute for Health Research (NIHR) [grant number MR/V035924/1] using UK aid from the UK Government to support global health research. The views expressed in this publication are those of the author(s) and not necessarily those of the MRC, NIHR, or the UK government.

Data availability statement:

Qualitative data are available upon reasonable request. All other documents reviewed for this article are publicly available.

Ethics Approval:

This study was approved by the Ethics and Scientific Review Committee at AMREF Health Africa in Kenya (ESRC P900-2020) and the National Institute for Medical Research (NIMR/HQ/R.8a/Vol.IX/3806) in Tanzania.

Competing interests:

None declared

For peer review only - http://bmjopen.bmj.com/site/about/guidelines.xhtml
For peer review only

References

1. World Health Organization. *WHO Coronavirus (COVID-19) Dashboard*. 2023 November 8th, 2023 [cited 2023 November 15th,]; Available from: https://covid19.who.int/.
2. Bouba, Y., et al., *The determinants of the low COVID-19 transmission and mortality rates in Africa: a cross-country analysis.* Frontiers in public health, 2021. **9**: p. 751197.
3. Formenti, B., et al., *The impact of COVID-19 on communicable and non-communicable diseases in Africa: a narrative review.* Le Infezioni in Medicina, 2022. **30**(1): p. 30.
4. Okonji, E.F., et al., *Understanding varying COVID-19 mortality rates reported in Africa compared to Europe, Americas and Asia.* Tropical Medicine & International Health, 2021. **26**(7): p. 716-719.
5. Haider, N., et al., *Lockdown measures in response to COVID-19 in nine sub-Saharan African countries.* BMJ Global health, 2020. **5**(10): p. e003319.
6. Mfinanga, S.G., et al., *Tanzania's position on the COVID-19 pandemic.* The Lancet, 2021. **397**(10284): p. 1542-1543.
7. Tessema, G.A., et al., *The COVID-19 pandemic and healthcare systems in Africa: a scoping review of preparedness, impact and response.* BMJ global health, 2021. **6**(12): p. e007179.
8. Kraef, C., et al., *The COVID-19 pandemic and non-communicable diseases—a wake-up call for primary health care system strengthening in sub-Saharan Africa.* Journal of primary care & community health, 2020. **11**: p. 2150132720946948.
9. Chudasama, Y.V., et al., *Impact of COVID-19 on routine care for chronic diseases: a global survey of views from healthcare professionals.* Diabetes & Metabolic Syndrome: Clinical Research & Reviews, 2020. **14**(5): p. 965-967.
10. World Health Organization - NCD Department, *Final Results: Rapid assessment of service delivery for non-communicable diseases (NCDs) during the COVID-19 Pandemic*. 2020.
11. Nikoloski, Z., et al., *Covid-19 and non-communicable diseases: evidence from a systematic literature review.* BMC Public Health, 2021. **21**(1): p. 1-9.
12. Richardson, S., et al., *Presenting Characteristics, Comorbidities, and Outcomes Among 5700 Patients Hospitalized With COVID-19 in the New York City Area.* JAMA, 2020. **323**(20): p. 2052-2059.
13. Vitiello, A. and F. Ferrara, *The impact of COVID-19 in diabetic patient.* Archives of Medicine and Health Sciences, 2020. **8**(1): p. 167.
14. Schofield, J., L. Leelarathna, and H. Thabit, *COVID-19: Impact of and on Diabetes.* Diabetes Therapy, 2020. **11**(7): p. 1429-1435.
15. Jordan, R.E., P. Adab, and K. Cheng, *Covid-19: risk factors for severe disease and death*. 2020, British Medical Journal Publishing Group.
16. Gilbert, M., et al., *Preparedness and vulnerability of African countries against importations of COVID-19: a modelling study.* The Lancet, 2020. **395**(10227): p. 871-877.
17. Sobngwi, E., et al., *Type 2 diabetes control and complications in specialised diabetes care centres of six sub-Saharan African countries: the Diabcare Africa study.* Diabetes research and clinical practice, 2012. **95**(1): p. 30-36.
18. Gill, P., et al., *Methods of data collection in qualitative research: interviews and focus groups.* British Dental Journal, 2008. **204**: p. 291.
19. Stemler, S., *An overview of content analysis.* Practical assessment, research, and evaluation, 2000. **7**(1): p. 17.

For peer review only - http://bmjopen.bmj.com/site/about/guidelines.xhtml

20. Ministry of Health - Tanzania, *Tanzania Health Policy (in Kiswahili)* 2007.
21. Kenya. Ministry of Health, *Kenya Health Policy 2014-2030*. 2014, Ministry of Health.
22. Ministry of Health, *The Kenya National Strategy for the Prevention and Control of Non-communicable Diseases - 2015-2020*. 2015.
23. Ministry of Health and Social Welfare, *Health Sector Strategic Plan (HSSP IV) July 2015 - June 2020*. 2015.
24. Government of the Republic of Tanzania, C.D. Ministry of Health, Gender, Elderly, and Children, *Strategic and action plan for the prevention and control of non communicable diseases in Tanzania 2016-2020*. 2016.
25. Ministry of Health -Community Develeopment, G., Elderly and Children,, *National Guideline of Clinical Management and Infection Prevention and Control of Novel Coronavirus (COVID-19).*
26. Kenyan Ministry of Health (MOH), *Interim Guidelines on Management of COVID-19 in Kenya*. 2020.
27. Kenya Ministry of Health (MOH), *Guidelines on Case Management of COVID-19 in Kenya*. 2021.
28. Ministry of Health - Office of the Director General, *Non-Communicable Diseases Clinic During COVID_19 Outbreak*. 2020.
29. Ministry of Health, C.D., Gender, Elderly and Children,, *Guidance on the provision of NCD and mental health services in the context of the covid-19 outbreak in Tanzania*. 2020.
30. Kenya Ministry of Health (MOH), *Interim Guidance on Continuity of Essential Health Services during the Covid-19 Pandemic.* 2020.
31. Kenya Ministry of Health (MOH), *Guidance on Continuity of Essential Health Services during the Covid-19 Pandemic*. 2020.
32. Kenya Ministry of Health (MOH), *Interim Guidance on Provision of Services for NCDs during the COVID-19 Pandemic*. 2020.
33. United Nations, *Flash appeal for Covid-19 Tanzania*. 2020.
34. Tanzania NCD Alliance (TANCDA), *Policy brief on the inclusion of NCDs on universal health coverage in Tanzania*. 2021.
35. Kenya Ministry of Health (MOH), *Kenya Emergency Medical Care Policy 2020 - 2030*. 2021.
36. Department of non-communicable diseases - Ministry of Health, *National Strategic Plan for the Prevention and Control of Non-Communicable Disease 2021/22 - 2025/26*. 2021.
37. Government of Kenya, *Kenya Vision 2030*. 2007.
38. Kassa, M.D. and J.M. Grace, *Noncommunicable Diseases Prevention Policies and Their Implementation in Africa: A Systematic Review.* Public Health Reviews, 2022. **42**: p. 1604310.
39. International Federation of Red Cross (IFRC) Disasater Law, *Law and public health emergency preparedness and response: Lessons from the COVID-19 pandemic*. 2021, International Federation of Red Cross and Red Crescent Societies. p. 174.
40. Kiragu, Z.W., et al., *Access to Medicines for Non-Communicable Diseases (NCDS) during COVID-19 in Kenya: A Descriptive Commentary.* Health Systems & Reform, 2021. **7**(1): p. e1984865.
41. Owopetu, O., L.-K. Fasehun, and U. Abakporo, *COVID-19: implications for NCDs and the continuity of care in Sub-Saharan Africa.* Global Health Promotion, 2021. **28**(2): p. 83-86.

For peer review only - http://bmjopen.bmj.com/site/about/guidelines.xhtml

42. Devi, R., et al., *The impact of COVID-19 on the care of patients with noncommunicable diseases in low-and middle-income countries: an online survey of patient perspectives.* Journal of Patient Experience, 2021. **8**: p. 23743735211034091.

43. Juma, P.A., et al., *Non-communicable disease prevention policy process in five African countries authors.* BMC Public Health, 2018. **18**(1): p. 961.

Figures

Fig 1: Search strategy

Tables

Table 1: Characteristics of Key Informants

Table 2: Description of the documents included in the desk review for Kenya and Tanzania

For peer review only

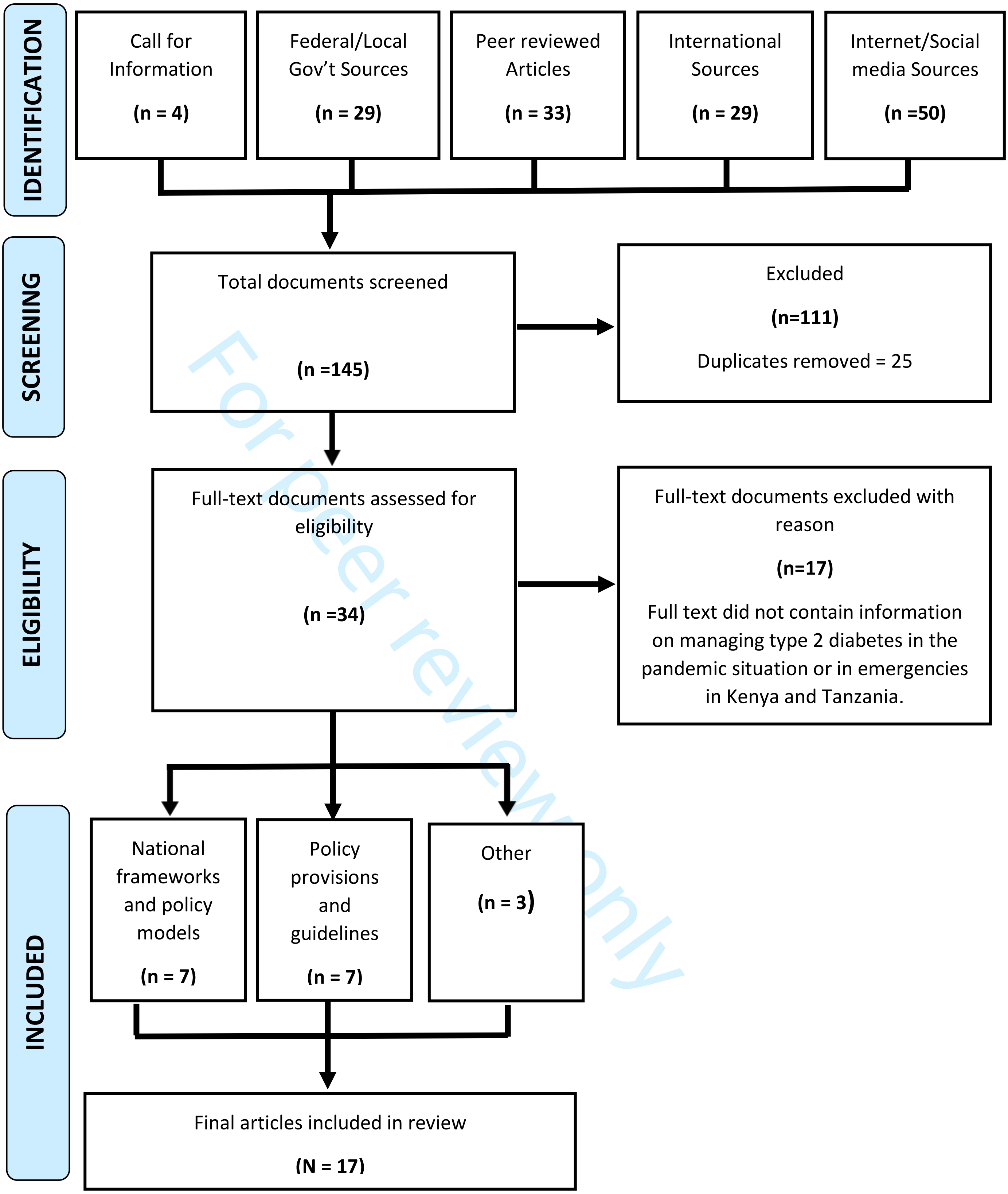

Figure 1: Search strategy

Appendices

Appendix A. Search Criteria for Peer-reviewed literature

1. **Diabetes terms:** diabetes, NCDs, disease management, Type 2 diabetes, diabetes mellitus, chronic disease, non-insulin dependent diabetes, ’type 2 diabetes’ OR ‘type II diabetes’ OR ’diabetes mellitus’ OR ‘diabetes’
2. **COVID terms**: covid, coronavirus, COVID-19, SARS-COV-2, severe acute respiratory syndrome coronavirus 2, 2019-nCoV
3. **Healthcare:** healthcare, disruption, impact, health services provision, change, continuity of care, medical care
4. **Policies**: policy, guidelines, measurement/assessment/evaluation, strategies, recommendations, plans, impact, research, outcomes, result, approaches, care continuity, SOPs, procedures, frameworks, policy models, laws, bills, tools
5. **Countries**: Kenya, Tanzania

For peer review only

Appendix B: Table 1: Details of search strategy

Source	Details
Call for information	A call for information was disseminated via email and social media platforms (WhatsApp) to relevant networks/experts including Ministry of Health officials, National NCD department heads, County health departments, diabetes clinic heads, National COVID-19 task force leads; Technical working groups (national or county), Academic researchers who focus on COVID/diabetes care disruption, and NGOs. We first identified a list of potential stakeholders from relevant networks and sent out the call to them. In the call we requested that they share hard or soft copy documents by email or courier related to the management of T2D and other chronic diseases during COVID-19 or other emergency epidemic or pandemic situations.
Peer reviewed literature databases	We searched peer reviewed literature databases of PubMed/Medline, Embase, Google Scholar, Web of Science, OpenGrey using search criteria to identify articles and grey literature that describe diabetes, COVID-19 and health system responses (*see specific search criteria in Appendix A*). Due to time limitations, this was not a comprehensive systematic review, but rather a rapid review of the literature.
Local/ International resources	Since international health agencies such as the World Health Organization have taken the lead both on COVID-19 responses and on management of NCDs, we reviewed documents from international resources, including the United Nations, World Health Organization, and relevant non-governmental agencies. We included WHO country profiles, WHO documents on COVID, and reports from NGOs working in diabetes in Kenya.
Policy documents/drafts from	We reviewed Kenya's health ministry websites including for all documents associated with national NCD strategies, policy documents and committee reports related to T2D and COVID-19, reports from meetings and events, and standard operating procedures. We searched websites including Ministry of health, and any COVID-specific working group, etc. for policies,

For peer review only

relevant national ministries	guidelines, measurement/assessment/evaluation, strategies, recommendations, plans, impact, research, outcomes, result, approaches, care continuity, SOPs, procedures, frameworks, policy models, laws, bills, tools.
Internet search	Given the extensive use of the internet to communicate during the pandemic, we conducted an internet search using google, Google Scholar and other search engines to identify patient support groups, diabetes association's/message boards, newspaper/media articles regarding T2D and COVID.
Social Media	Finally, given the speed with which governments needed to react to COVID-19 developments, many governmental individuals and offices disseminated information to the public on social media. We reviewed twitter pages of the country's Ministry of Health and other high-profile individuals/offices, presidential speeches or other speeches on social media and national NCD groups for information about T2D and COVID-19.

For peer review only

Appendix C: Key Informant Interview guide for policy actors working in non-communicable diseases (NCDs).

GECO Study

Project Title: Healthcare and Socio-economic Impacts of COVID-19 on Patients with type 2 Diabetes in Kenya

Key Informant Interview (KII) guide – Policy Actors working in non-communicable diseases (NCDs).

AFRICAN POPULATION AND HEALTH RESEARCH CENTRE

For peer review only

These are the interviews with policy actors and decision makers working in non-communicable diseases and other related fields. The objective is to identify policy gaps in Kenya to inform policy change, priority setting and action for T2D management during COVID-19

Introduction and Warm up

Moderator Note: Moderator to read out from written informed consent form and obtain signature/ thumbprint)

- The moderator introduces him/herself and explains the purpose of the study.
- Collects relevant information on the Participant Description Form (PDF) during screening.
- The moderator explains that the discussion is open, not an exam and there is no right or wrong answer. The facilitator/moderator should explain to the respondent that the information given by the participant is confidential.
- Encourage the respondent to give honest opinions.
- Explain the use of the recorder.
- Start tape recording if consent is granted: (Moderator to switch recorder on)

Remember to introduce each section to the participant to prepare them for the questions to be asked.

For peer review only

This study aims to identify policy gaps in Kenya to inform policy change, priority setting and action for T2D management during COVID-19. In particular, we would like to know what policies/guidelines have been developed during COVID-19, who and which sectors have been involved in the policy development and implementation, challenges in the formulation and implementation, gaps that still exist and recommendations. We are interested in higher level policies such as laws, regulations, national strategic plans, directives as well as lower level guidelines, action plans, SOPs etc. related to T2DM during COVID-19.

Section A: General response of governments to COVID-19

1. Briefly tell me what the government's response has been towards the management of NCDs including type 2 diabetes during COVID-19 in Kenya?

Section B: Policy formulation during COVID-19

2. Please tell me about the policies and guidelines that you are aware of that were **formulated** during COVID-19 to manage people with type 2 diabetes

   Probes:

   Context

   a) What was the rationale for the formulation of the policy/guidelines mentioned? (context)
      i. What is it about the COVID-19 situation that necessitated the development of the policy/ guideline?
      ii. Was there any research that informed the need to update/revise the pre-existing NCD/diabetes policies/guidelines? Please explain.

   Content

   b) What do the policies/guidelines include? (content)
      i. Were the provisions (content) in the policy adequate to manage type 2 diabetes during COVID-19? If not, what else do you think should be added?
      ii. Was the content in the policy/guideline informed by research findings?

   Actors and Process

   c) Were you involved in the formulation of these policies you have mentioned? (actors)
   d) What was your role in the formulation?
   e) Who else or what other sectors were involved in the formulation of the policy or guidelines?
      i. Who led the process of formulating the type 2 diabetes policies or guidelines?
      ii. What was the role of the sectors involved?
      iii. Who else should have been involved in the formulation?
         i. Were healthcare providers involved in the formulation?
   f) What were the advantages of having these policies/guidelines in place during COVID-19?
   g) What were the challenges encountered during the formulation process of the policy/ guidelines for type 2 diabetes?

3. What resources were available for the **formulation** of these policies/guidelines?

   Probes:

   a) Type of resources available? (funding, human resource, experts in the field)

Section C: Policy implementation during COVID-19

4. To what extent have the policies/guidelines developed during COVID-19 been **implemented**

Probes:

a) Were you involved in their implementation? (actors)
b) What was your role in the implementation?
c) Who else or what other sectors were involved in the implementation of the policy/guidelines?
    i. Who led the implementation of the policies/guidelines?
    ii. What was the role of the sectors involved?
    iii. Who else should have been involved in the implementation? Why?
d) What were the advantages of implementing these policies/guidelines during COVID-19?
e) What were the challenges encountered during the implementation of the policy/guidelines?

5. What resources were available for the **implementation** of these policies/guidelines?

Probes:

a) Type of resources available? (funding, human resource, experts in the field)
b) Were the resources adequate? What was missing? Elaborate

6. What mechanisms were used to disseminate the new policies/guidelines on the management of type 2 diabetes during COVID-19?

Probes:

a) Types of mechanisms? (trainings, directives shared etc?)
b) How does the government ensure that the policies/guidelines are actually being used?

Section D: Recommendations

7. What gaps still exist in the management of type 2 diabetes during COVID-19?
    a) How can they be addressed to avoid future disruption in care during an emergency/pandemic situation?
8. In your opinion, how best can the Kenyan health system **strengthen its response** towards chronic disease management in the event of an emergency/pandemic situation?
9. What would you recommend to ensure the continuity of chronic care management (including type 2 diabetes) during an emergency/pandemic situation?
10. Is there anything else you would like to add with regard to chronic care management including diabetes in Kenya that we have not discussed?

We have come to the end of the interview. ***[Thank the participant for participating in the interview]***

*****END*****

For peer review only

Appendix D: Description of the documents included in the desk review for Kenya and Tanzania

Type of Document	Name of Document	Country	Dates	Content Description
1. Policy [12]	Tanzania Health Policy 2007	Tanzania	2007	The policy provides direction on the development of NCD guidelines that will provide guidance on the provision of health service, equity in service provision, capacity building, and raising awareness on the management and prevention of NCDs.
2. Policy [13]	Kenya Health Policy 2014-2030	Kenya	2014	The document provides overall directions to ensure significant improvement in the overall status of health in Kenya, the country's long-term development agenda, Vision 2030 and global commitments including halting and reversing the rising burden of NCDs in the country.
3. National Framework [14]	National Strategy for the Prevention and Control of NCDs 2015 -2020	Kenya	2015 - 2020	The document emphasizes an integrated approach towards addressing NCDs in order to reduce the preventable burden, avoidable morbidity, mortality, risk factors, and costs due to NCDs. It provides evidence based NCD prevention and control interventions to ensure optimal health throughout the life course for sustainable socioeconomic development.
4. National Framework [15]	Health Sector Strategic Plan (HSSP IV)	Tanzania	2015-2020	The document clarifies the importance of integrating NCD clinics into the health care system to enhance access to care. It also describes NCD prevention and early detection of the NCD and start of medication.
5. National framework [16]	Strategic and action plan for the prevention and control of NCDs in Tanzania 2016 – 2020	Tanzania	2016-2020	The document advocates for NCD prevention and control at a national level and it emphasizes strengthening leadership, governance, multisectoral collaboration, and accountability for the prevention and control of NCDs and promoting preventive curative and rehabilitative services.

For peer review only

6. Guideline [17]	National guideline of clinical management and infection prevention and control of novel coronavirus (COVID-19)	Tanzania	April 2020	This guideline educates the public on COVID-19 preventive measures and emphasizes the provision of accurate information for people with NCDs to protect them from COVID-19.
7. Guideline [18]<br><br>8. Guideline [19]	Interim Guidelines on Management of COVID-19 in Kenya<br><br>**Updated version:** Guidelines on Case Management of COVID-19 in Kenya	Kenya	April 2020<br><br>July, 2021	The guidelines combine both preventive and clinical management of diseases during COVID-19. The updated version (2021) contains the latest guidelines for the clinical management of COVID-19 and includes case definitions, infection prevention and control, diagnosis and management of COVID-19, and ending isolation for COVID-19 patients.
9. Letter [20]	Non-Communicable Diseases Clinic During COVID-19 Outbreak	Kenya	Mid-April 2020	This document is a national directive for NCD clinics to remain operational in all counties during the COVID-19 pandemic.
10. Guideline [21]	Guidance on the provision of NCD and mental health services in the context of the COVID-19 outbreak in Tanzania	Tanzania	May 2020	This guideline targets strengthening NCD service provision during COVID-19 including capacity building for health workers, health promotion for NCD/T2D patients, health facility re-structuring for COVID-19 prevention, and access to care and continuity of essential health services.
11. Guideline [22]<br><br>12. Guidelines [23]	Interim Guidance on Continuity of Essential Health Services during the Covid-19 Pandemi**c.**<br><br>**Updated version:** Guidance on Continuity of Essential Health Services	Kenya	May 2020<br><br>July 2020	These documents provide guidance for healthcare providers on immediate actions that should be considered to reorganise the health system to ensure continuity of health services during the COVID-19 pandemic for all services including NCD services.

	during the COVID-19 Pandemic			
13. Guideline [24]	Interim Guidance on Provision of Services for NCDs during the COVID-19 Pandemic	Kenya	July 2020	The document highlights specific COVID-19 related challenges on NCDs including severity among high-risk persons, COVID-19 actions/responses jeopardising access to care and disrupting lifestyle approaches, disruption of funding and supplies, and the general management of NCDs.
14. Report [25]	Flash appeal for COVID-19 Tanzania	Tanzania	July – December 2020	The document discusses strengthening capacity building for health workers, ensuring continued mentorship on NCD and COVID-19 health management, and disseminating information materials and guidelines. It also recommends health promotion and public health intervention strategies for quality-of-life improvement for those with complications associated with NCDs.
15. Policy Brief [26]	Tanzania NCD Alliance (TANCDA) policy brief on the inclusion of NCDs on universal health coverage in Tanzania	Tanzania	March 2021	The document recommends various actions to ensure access to quality NCD care, appropriate health financing strategies, multisectoral collaboration on prevention and control of NCDs, and health awareness creation during the COVID-19 pandemic.
16. Policy [27]	Kenya Emergency Medical Care Policy 2020 – 2030	Kenya	July 2021	This document was developed to provide guidance including necessary structures, resources, regulations and standards needed to establish an emergency medical care system in Kenya. It aims to reduce morbidity and mortality caused by medical emergencies, including those emergency conditions caused by NCDs.
17. National framework [28]	National Strategic Plan for the Prevention and Control of Non-Communicable Disease 2021/22 - 2025/26	Kenya	August 2021	The document provides direction on strengthening sectoral and multi-sectoral coordination, leadership and governance for prevention and response to NCDs at all levels. It highlights the importance of sustainable NCD management, promotion of NCD research, and strengthening surveillance, monitoring and evaluation of NCDs to inform decision making and health planning.

For peer review only
